# High‐Concentration Alcohol Generation in Bipolar Membrane CO Electrolyzer

**DOI:** 10.1002/anie.202517470

**Published:** 2025-11-18

**Authors:** Wenjin Zhu, Qiu‐Cheng Chen, Yiqing Chen, Jianan Erick Huang, Guangcan Su, Hengzhou Liu, Weiyan Ni, Yuanjun Chen, Jiaqi Yu, Bosi Peng, Jiantao Li, Sungsik Lee, Shaoyun Hao, Yuxia Shen, Huajie Ze, Bei Zhou, Xiao‐Yan Li, Yali Ji, Shuang Yang, Cong Tian, Yongxiang Liang, Ke Xie, Edward H. Sargent

**Affiliations:** ^1^ Department of Chemistry and Department of Electrical and Computer Engineering Northwestern University Evanston Illinois USA; ^2^ X‐ray Science Division Argonne National Laboratory Lemont Illinois USA

**Keywords:** CO electroreduction, Forward‐biased bipolar membrane, Highly concentrated alcohols production

## Abstract

Electrochemical reduction of carbon dioxide and carbon monoxide offers an electricity‐powered route to make multicarbon liquid products. However, in conventional systems employing anion exchange membranes (AEMs), significant liquid product crossover leads to dilute product streams, increasing separation costs; and also produces unwanted anodic oxidation, further decreasing overall efficiency. Here, we report a forward‐biased bipolar membrane (FB‐BPM) system that achieves <10% liquid product crossover while sustaining a highly alkaline environment near the cathode, suppressing ethylene and hydrogen and favoring liquid products. By tuning catalyst composition to modulate the adsorption of *H and *OH, we steer selectivity toward acetate and alcohols. Using the FB‐BPM system, we achieve >25 wt% acetate on CuZn and >15 wt% alcohols on CuSn directly from the cathode outlet stream.

## Graphical abstract

This study presents a forward‐biased bipolar membrane (FB‐BPM) CO electrolyzer that reduces liquid product crossover to <10% (8x lower than conventional designs) while minimizing hydrogen production and in favor of multicarbon liquid species. By engineering catalyst morphology to modulate the binding of *CO, *H, and *OH intermediates, we identified a CuSn catalyst enhancing C2–C1 coupling pathways for alcohols formation. Integrating the reactor design with catalyst innovation, we achieve >15 wt% alcohols production directly from the cathodic outlet.

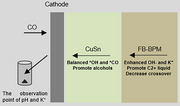

## Introduction

Electrochemical reduction of carbon dioxide (CO_2_R) produces acetic acid, ethanol and N‐propanol^[^ ^1^, ^2^, ^3^, ^4^ ^]^ and many efforts have been dedicated to achieving high Faradaic efficiency (FE) and low cell voltages.^[^ ^5^, ^6^, ^7^ ^]^ In anion exchange membrane (AEM)‐based CO_2_R systems, the strong local alkaline environment required to produce multicarbon liquid products leads to carbonate/bicarbonate formation, limiting carbon utilization and adding energy cost for CO_2_ regeneration.^[^ ^8^, ^9^, ^10^, ^11^ ^]^

To avoid carbonate issues, an alternative is a cascade reaction starting from CO_2_R to CO in a solid‐oxide electrochemical cell ‐ a device not susceptible to carbonate formation; this is followed by carbon monoxide reduction (COR) to liquid hydrocarbons. This tandem system has shown promising energy consumption compared to direct CO_2_R^5^.^[^ ^12^ ^]^

Electrochemical CO‐to‐liquid conversion is challenged by product crossover and lack of a highly selective and stable cathode catalyst. In traditional AEM systems, electro‐osmotic drag and concentration polarization cause substantial crossover of liquid products from the cathode to the anode. This phenomenon complicates the collection of concentrated products and increases downstream separation costs.^[^ ^13^ ^]^ To address this, researchers have explored alternative configurations, such as replacing AEM with bipolar membranes (BPM)^[^ ^14^ ^]^ and cation exchange membranes (CEM).^[^ ^15^ ^]^

We focus here on alcohols – portable liquids having a high energy density – as the product. Much work has already been done studying how catalyst composition impacts alcohol selectivity; we sought to study further the interplay between catalyst and membrane. There are indications that this interplay is important: copper nitride doped with gold nanoparticles and isolated silver atoms was reported to increase the carbophibicity of the catalyst surface, providing C2 + alcohol FE of > 70%;^[^ ^16^ ^]^ a dilute alloy catalyst was reported to stabilize the relevant C1 and C2 intermediates via proximate active sites, and to provide n‐propanol electrosynthesis from carbon monoxide with FE47 ± 3%.^[^ ^17^ ^]^ In the present work, we examine further membrane: catalyst codesign.

### Comparison of AEM, BPM Systems for High Liquid Product FE and Low Crossover Rate

**Figure 1:**
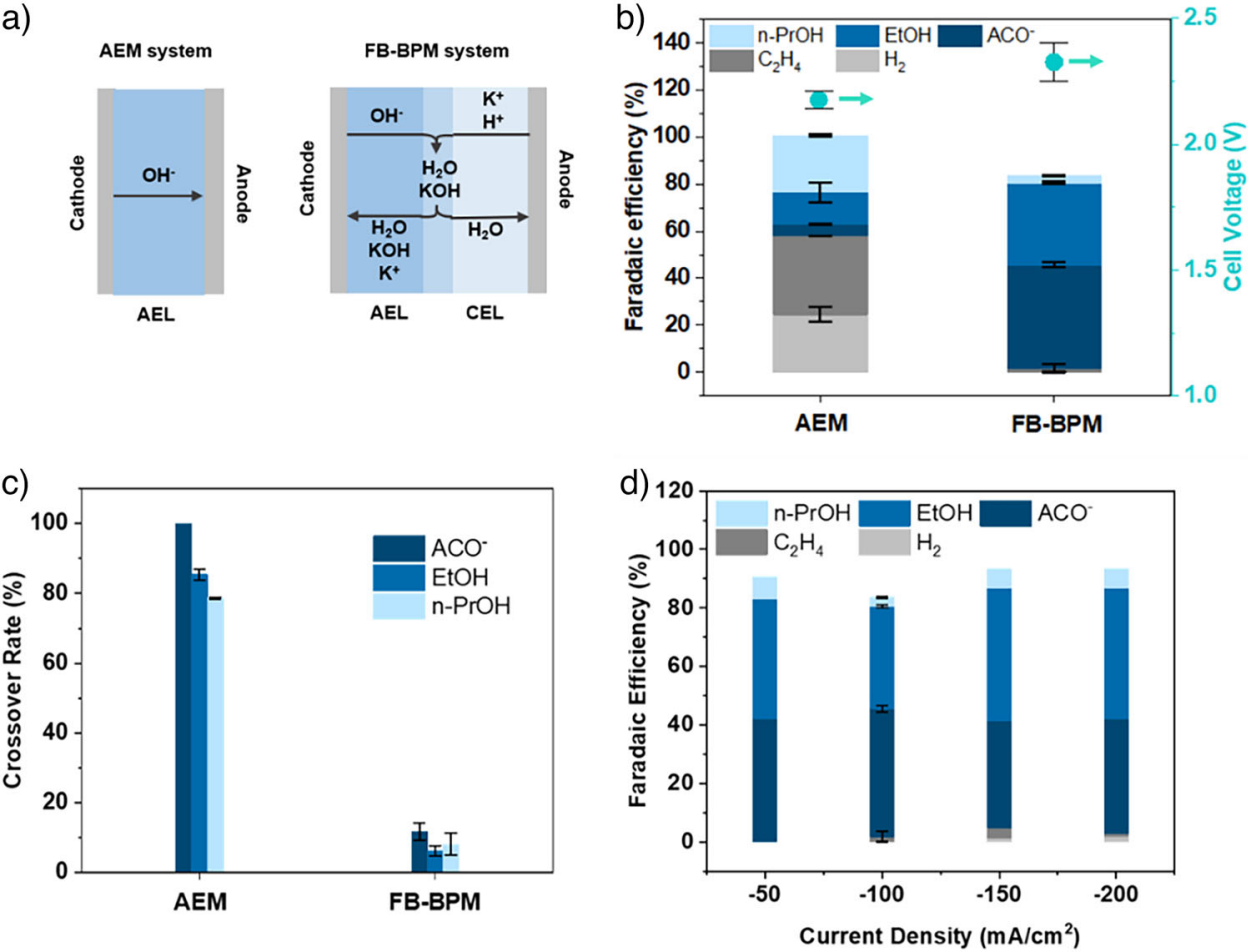
a) Anion exchange membrane (AEM) and forward‐bias bipolar membrane (FB‐BPM) systems. b) Faradaic efficiency and cell voltage of Cu catalysts toward COR, with AEM and FB‐BPM. c) Crossover rates of acetate, ethanol, n‐propanol on Cu catalysts toward COR, with AEM and FB‐BPM. d) Faradaic efficiency of Cu catalysts toward COR with FB‐BPM under different current densities.

The problem of liquid product crossover remains an issue to be tackled in AEM systems (Figure 1a). We baselined the distribution of CORR products on cathode and anode as a function of the membrane, in each case using a Cu catalyst (Figures S1–S3) and operating at −100 mA/cm^2^ (methods are shown in Figures S4–S7). In AEM systems, OH^−^ generated on the cathode passes through the membrane to recombine with H^+^ generated on the anode; fast water transport through the membrane (Piperion and Sustainion) provides alkali cations from the anolyte, achieving a balanced cation and local pH environment on the cathode for C─C coupling to C2+ products. When we worked with 50 um AEM, gaseous products (H_2_ + C_2_H_4_) make up 58% of total faradaic efficiency, with the other 42% of the FE attributable to C2 + liquids (Figure 1b). However, we observed that 80–90% of liquid products generated in electrosynthesis at the cathode ended up in the anolyte, the result of crossover (Figure 1c). The less dense microstructure of the AEM allows facile liquid transport. Since electro‐osmotic drag^[^ ^18^ ^]^ due to the applied bias across the cell may play a significant role in crossover (Figures S8 and S9), we used a CEM to reverse the ion‐migration direction. When we used a 50 um CEM, liquid product crossover was suppressed to 25%. In this system, total gas:liquid products were in a ratio ∼ 1:2 (Figure S10), consistent with a high alkali cation concentration and local alkalinity due to K^+^ transport from the anode to the cathode and the accumulation of OH^−^ at the cathode. A high local pH could suppress the competing HER; and high K^+^ concentration in the surface double layer tends to stabilize the O in the intermediate of COR prevents the formation of ethylene and fosters the ethanol and acetate pathway.^[^ ^19^, ^20^, ^21^, ^22^ ^]^

We studied how reverse versus forward BPM impacted COR selectivity and crossover. In the reverse‐bias (RB‐BPM) configuration, where the CEL is proximate to the cathode, H^+^ dissociated from H_2_O at the AEL:CEL interface migrates to the cathode, and OH^−^ to the anode (Figures S11 and S12). This configuration leads to a less locally alkaline environment, which favors the hydrogen evolution reaction (HER) and less total liquid product.^[^ ^23^ ^]^ The voltage penalty due to the water splitting layers leads to a high cell voltage of −3.2 V (Figure S13). In contrast, FB‐BPM system consisting of 50 um AEL and 50 um CEL to avoid the high voltage associated with the water dissociation, showing a cell voltage similar to that of pure AEM and pure CEM system (Figure 1b). We found the FB‐BPM surface resistivity to be 1.5 Ω·cm^2^, measured using electrochemical impedance spectroscopy (EIS). In FB‐BPM, we found that 82% of total FE went to C2 + liquids. When we ran each electrolyzer for 1 h, we observed dramatic differences in crossover rates (Figure 1c): >80% liquid crossover for AEM and < 10% liquid crossover for FB‐BPM. We evaluated the contributions of electro‐osmotic drag and concentration polarization to ethanol crossover (Figures S8 and S9). In the AEM‐based electrolyzer, electro‐osmotic drag was found to dominate over concentration polarization as the primary cause of ethanol crossover. The FB‐BPM design diminishes crossover associated with electro‐osmotic drag (Figure S9) and reduces concentration polarization. We propose a model (Figure 1a) of this phenomenon: the net flow of ions and molecules is directed toward the cathode, and the electro‐osmotic drag force pulls neutral molecules backward toward the cathode; and the migration of K⁺ ions to the cathode carries water with it, diluting the product concentration at the cathode. This dilution reduces the concentration‐driven driving force for liquid product crossover. In addition, the FB‐BPM is physically thicker, being the sum of an AEM and a CEM, providing a greater geometric barrier to crossover.

When we compared voltages in detail, we found that the full cell voltage of FB‐BPM is −0.1 V higher than that of the AEM system, assignable to a larger pH gradient,^[^ ^24^ ^]^ since the added AEM layer between the cathode and CEM increases the local pH near the cathode. This increase in local pH may further suppress the H_2_ and C_2_H_4_ FE and increase the liquid product FE. We compared the performance of different alkali cations (Li^+^, Na^+^, K^+^, and Cs^+^), and found K^+^ to show the highest C2 + liquid productivity (Figure S14). In this FB‐BPM system, we collect continuously a stream of liquid products from the cathode gas outlet, finding this to have 4 wt% ethanol and < 1 wt% propanol when we use Cu (Figures S15 and S16).

### Catalyst Screening to Improve Alcohol Selectivity

We observed a pronounced increase in Faradaic efficiency, from 40% to 80%, when we replaced the AEM with an FB‐BPM. A ∼20% deficit in total Faradaic efficiency may be because the liquid product is draggied, via electro‐osmosis, to the AEM/CEM interface, is trapped there, and can neither be detected on the cathode nor in the anode. The dopants exhibited a distinctive impact on COR selectivity compared to their behavior in AEM‐based electrolyzers. These findings motivated us to investigate how the FB‐BPM alters the local microenvironment and its resulting effects on reaction pathways. In the FB‐BPM system, a surface enriched in K⁺ and OH^−^^[^ ^13^, ^14^ ^]^ is expected to enhance OH* adsorption and strengthen the local electric field due to the high concentration of alkali cations, while simultaneously suppressing H* adsorption. This combination establishes a microenvironment favorable for liquid‐product formation and calls for new catalyst design principles tailored to FB‐BPM electrolyzers.

While the total multicarbon liquid FE was increased in the K^+^‐FB‐BPM system, acetate and alcohols were in similar proportion with one another. We note that the energy cost to separate acetate and regenerate KOH is estimated to be higher than that to separate ethanol at a similar concentration (Figure S17); and that alcohol has a higher energy density and larger market volume. We therefore pursued studies aimed at increasing the alcohol concentration in the cathode gas channel.

OH^*^ and H^*^ surface binding energies, influenced by the addition of metal dopants that change the oxophilicity of Cu sites, have been used to tune selectivity among liquid products.^[^ ^21^, ^25^, ^26^, ^27^, ^28^, ^29^ ^]^ We screened alloys that we synthesized by reducing Cu_x_M_y_O_z,_ to Cu‐M. We chose different metals from most to least oxophilic.^[^ ^30^ ^]^

**Figure 2:**
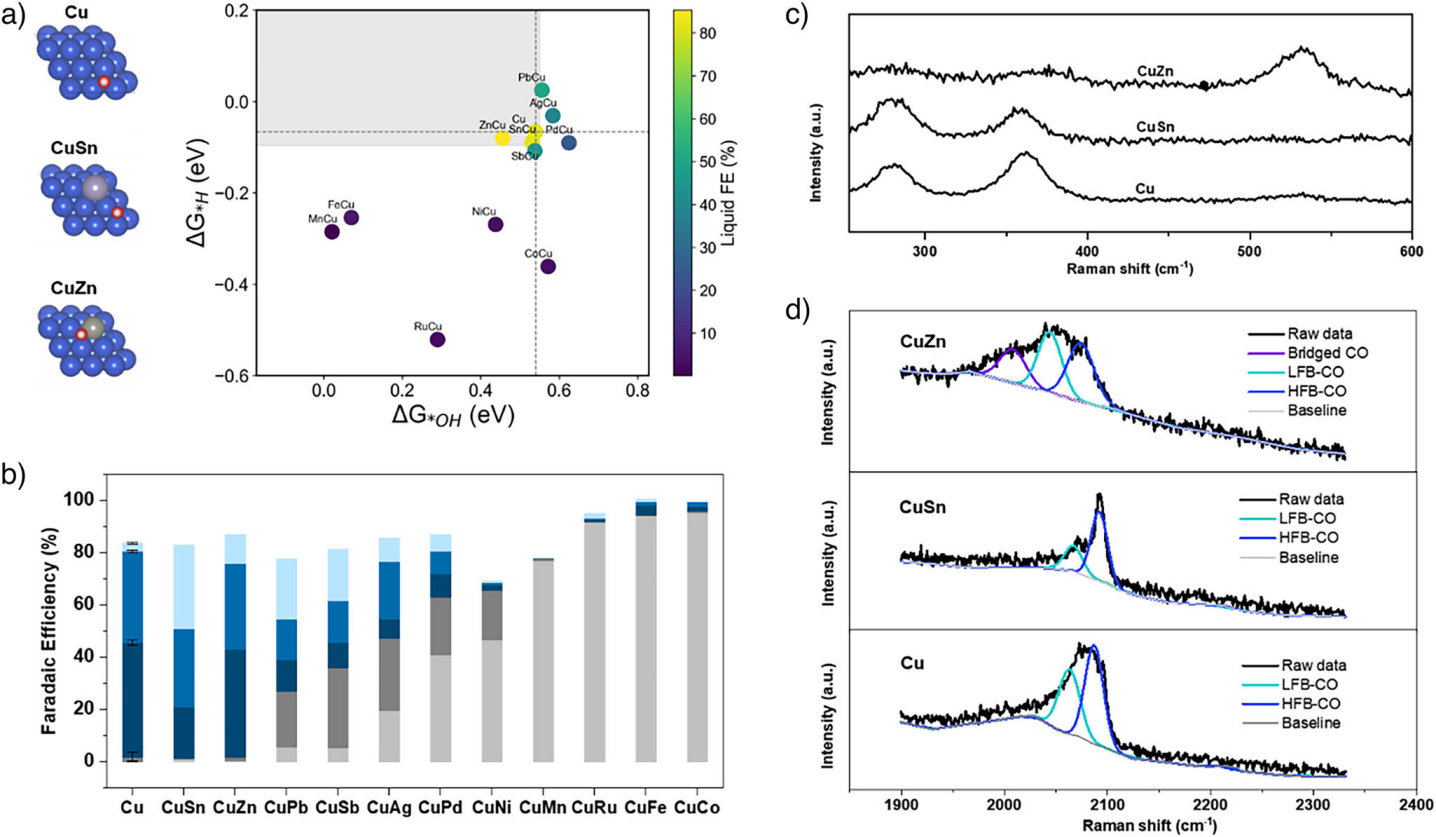
a) Gibbs free energy of hydrogen (∆G_*H_) and hydroxyl (∆G_*OH_) adsorption on Cu(111) and metal‐doped Cu(111) surfaces. The color map represents the liquid Faradaic efficiency from experiment, while the black dashed lines denote the adsorption energies of pristine Cu(111) used for reference. The grey‐shaded region highlights the optimal adsorption window for CORR to liquid products, achieved by maximizing the ∆G_*H_ and minimizing the ∆G_*OH_, defined by ∆G_*OH_ < ∆G_*OH‐Cu_ + 0.01 eV and ∆G_*H_ > ∆G_*H‐Cu_ ‐ 0.01 eV. b) Faradaic efficiency of Cu‐M alloy catalysts in FB‐BPM system toward COR. Cu, CuSn, CuZn show the highest liquid products Faradaic efficiency, corresponding well with the DFT speculations, as shown in the grey‐shaded region in Figure 2a. c) In situ Raman spectra for catalysts toward COR in the range of 200–600 cm^−1^ d) 1900–2300 cm^−1^. LFB‐CO: low‐frequency band linear CO; HFB‐CO: high‐frequency band linear CO.

For the catalysts we studied (Figure 2a,b, Figures S18–S23), the dopants showed similar trends in FB‐BPM and AEM systems on liquid products. We note that similar compositions have been reported in literature, and the faradaic efficiency trends appear to vary significantly among reports.^[^ ^31^, ^32^, ^33^ ^]^ We offer that future studies of the detailed impact of particle size and precursor effects could shed further light on the origins of catalytic performance. CuZn and CuSn were particularly effective in increasing liquid products. Zn dopants increased acetate (Figures S24–S30); while Sn dopants increased n‐propanol selectivity from less than 5% to over 30% FE. In particular, Sn‐doped Cu led to a total alcohol FE of 62%, with liquid products totaling 82% FE.

In the FB‐BPM system, a surface rich in K^+^ and OH^−^ is expected to facilitate the adsorption of OH*, and to produce a stronger local electric field in light of the high concentration of alkali cations; all the while reducing adsorption of H*,^[^ ^21^ ^]^ conditions known to suppress H_2_ and C_2_H_4_ and to favor the production of acetate. To study this further, we used DFT versus *OH and *H adsorption across catalyst compositions (Figure 2a, Figures S18–S20). We connect a high liquid Faradaic efficiency with catalysts that exhibit both high ∆G*H (indicative of minimized H* adsorption) and low ∆G*OH (indicative of maximized *OH adsorption). Tuning H* will cause a shift in selectivity between alcohols and acetate in the FB‐BPM system. As we change ∆G*H, a metal dopant that reduces the H* adsorption energy, such as CuSb, is expected to prefer ethylene, with a further reduction ultimately leading to hydrogen evolution. Varying the surface binding of OH* changes the selectivity between acetate versus ethylene (CuZn versus CuPd), where stronger OH* binding favors acetate. The highest alcohol selectivity achieved using Sn doped Cu, which is between the CuZn and CuPd, was achieved with a balanced combination of H* adsorption and OH*. Compared to Cu, CuSn exhibits a slightly stronger OH* and H*.^[^ ^17^ ^]^

To further understand how metal doping influences product selectivity, we conducted in situ Raman spectroscopy to investigate the changes of surface species on Cu, CuZn, and CuSn catalysts (Figure 2c–d). These spectra were collected at potentials ranging from −0.4 to −0.6 V versus RHE, where the surface CO coverage reached its maximum. The peaks centered at ∼280 cm^−1^ and ∼360 cm^−1^ correspond to the rotational and stretching vibrational modes of Cu–CO, respectively. A peak near 530 cm^−1^ is attributed to the Cu–OH* stretching mode. Notably, CuZn exhibits a much more pronounced Cu–OH* signal compared to Cu and CuSn, indicating a significantly higher OH* surface coverage (Figure 2c), an observation consistent with computational results that predict stronger OH* binding on CuZn surfaces.

The band at 2000–2100 cm^−1^ region (Figure 2d), representing the C≡O stretching vibrations of adsorbed CO, was deconvolved into three distinct components, corresponding to CO adsorption on bridge, terrace (LFB‐CO), and low‐coordination sites (HFB‐CO). CuZn displays the highest intensity for CO adsorption on terrace sites, whereas CuSn shows a greater proportion of CO bound to low‐coordination Cu atoms. CO adsorbed on terrace sites has been widely associated with enhanced C–C coupling. However, given that our reaction environment—rich in K⁺ and OH^−^—achieves over 95% selectivity toward C_2_⁺ products on all three catalysts, overcoming the C–C coupling step itself is of less interest, while the distribution of CO adsorption sites can serve as an indicator of product branching. Based on the performance and Raman results, we proposed that predominant CO adsorption on terrace sites, with closely packed CO on the surface, is likely to favor the production of acetate, which is consistent with prior reports that high CO coverage facilitates the acetate formation.^[^ ^34^, ^35^ ^]^ In contrast, a balanced distribution between terrace and low‐coordination could promote further hydrogenation and C_2_–C_1_ coupling pathways, such as those leading to propanol.

### Maximizing Alcohol Production and Concentration on CuSn

**Figure 3:**
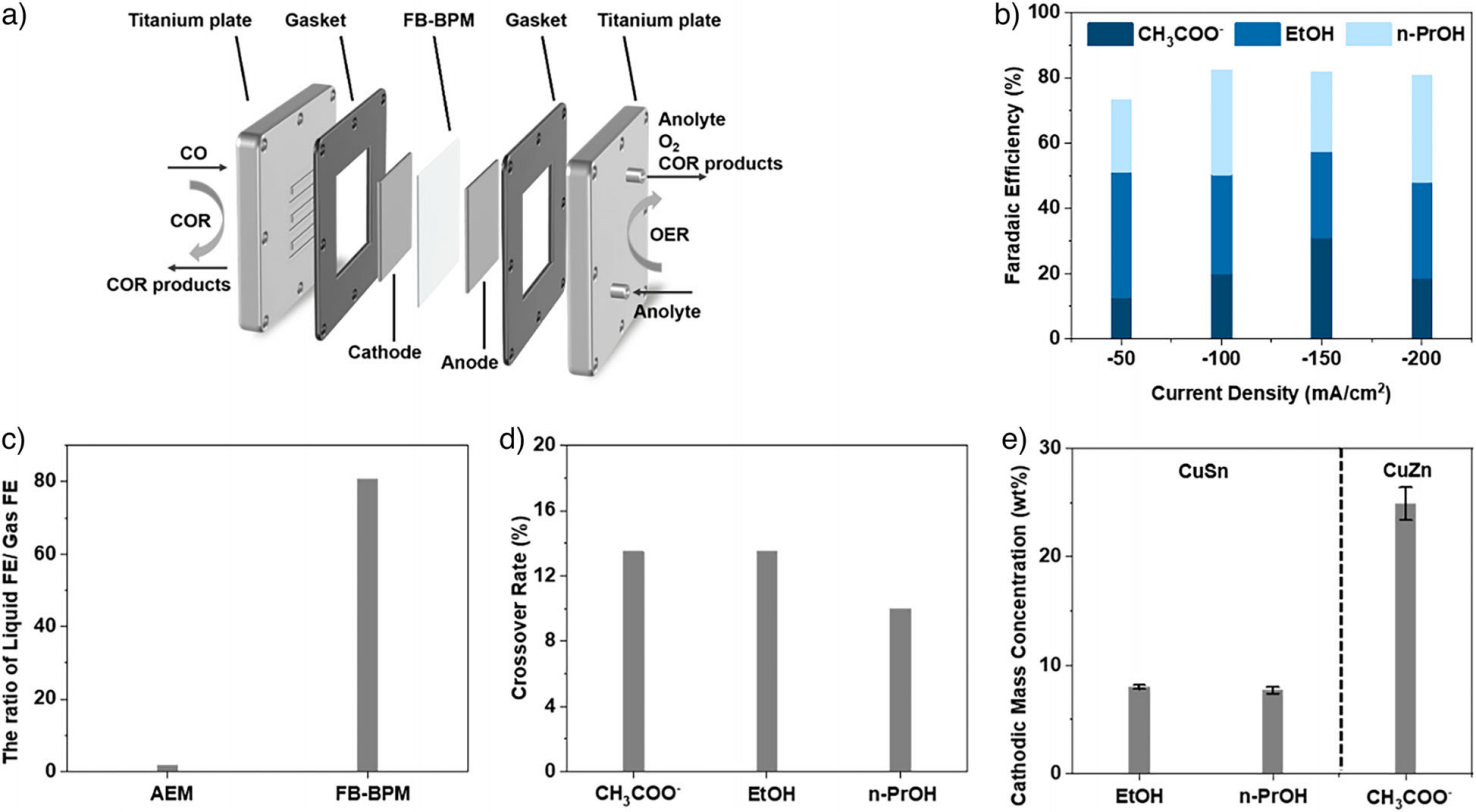
a) Schematic illustration of FB‐BPM system toward COR. b) Faradaic efficiency of liquid products for CuSn at different current densities in the FB‐BPM system. c) The ratio of liquid FE / gas FE for CuSn catalysts toward CO reduction, in AEM and FB‐BPM system, at the fixed current density of −100 mA/cm^2^. d) Crossover rates of acetate, ethanol, n‐propanol of CuSn toward COR in the FB‐BPM system. e) The left panel is the instant mass percentage (production rate) of alcohols at the cathode outlet, for CuSn in FB‐BPM system, at −100 mA/cm^2^. The right panel is the mass concentration of acetate from the cathodic outlet channel at −100 mA/cm^2^ in K^+^‐FB‐BPM system, with optimized CuZn catalyst (∼2 wt% Zn).

When we optimized CuSn (Figures S31–S34), we achieved 62% FE to alcohols at −200 mA/cm^2^, evenly divided between ethanol and n‐propanol (Figure 3b). The K⁺‐FB‐BPM system also demonstrated a ratio of total Faradaic efficiency for gas products to liquid products ∼80%, which is 70 times higher than that of the K⁺‐AEM system (Figure 3c). We measured a crossover rate of liquid products from cathode to anode of 14% for acetate and for ethanol, and 10% for n‐propanol (Figure 3d). We note though that ethanol may undergo oxidation to acetate at the anode. During stability tests, we observed that K^+^ was nearly undetectable in the cathode outlet stream during the 30–40 min period. During this phase, a concentrated alcohol stream (>15 wt%) was collected from the cathode outlet (Figure 3e). The condensate flow rate at the cathode outlet was derived from the product analysis. Given an n‐propanol concentration of ∼8 wt% in the condensate and a measured production rate of ∼5.5 mg/h, the total mass flow rate was calculated to be approximately 69 mg/h. Assuming a density of ∼1 g/mL, this is equivalent to a volumetric flow rate of ∼69 µL/h.

**Figure 4:**
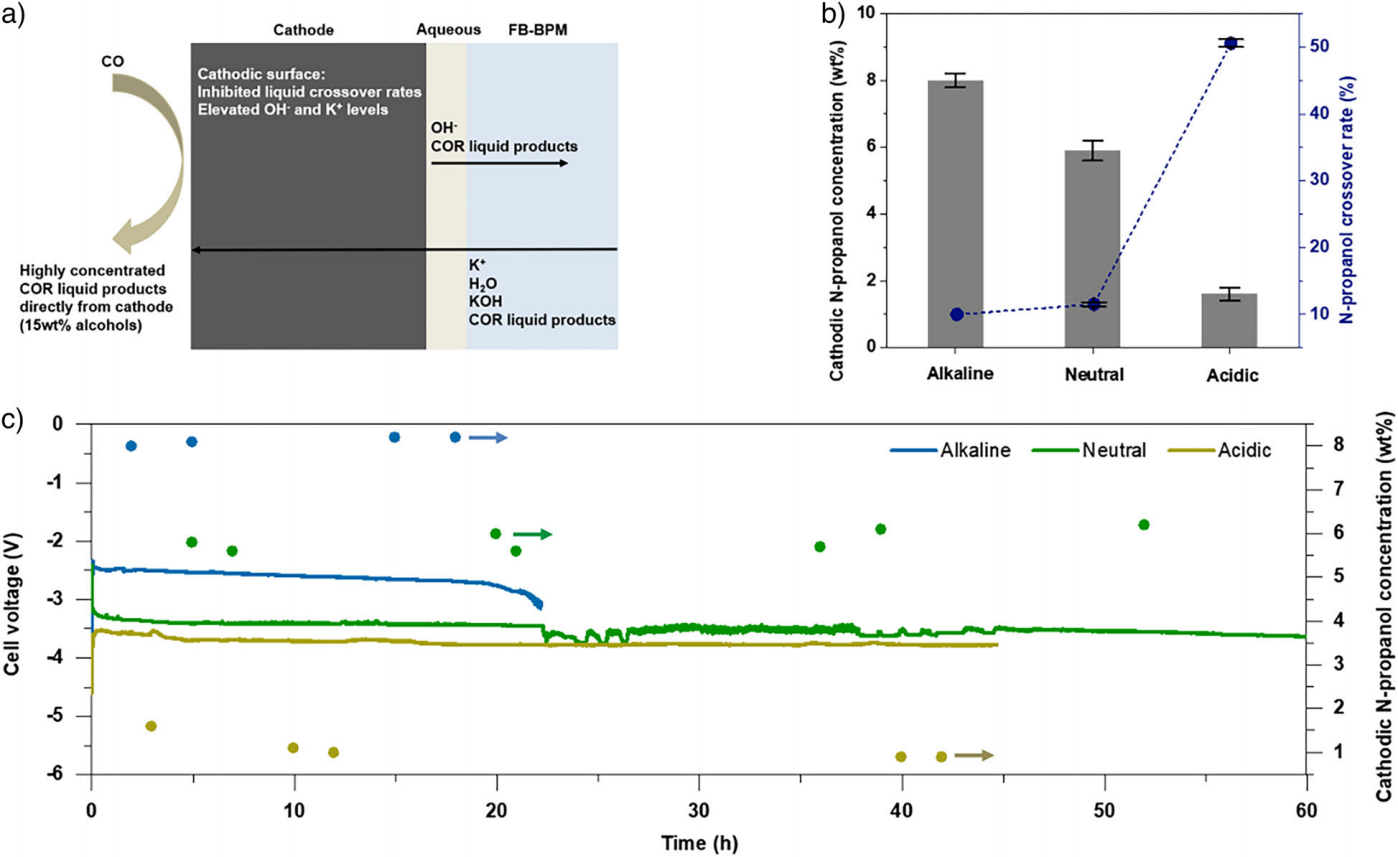
(a) Schematic illustration of the mechanism leading to the accumulation of highly concentrated alcohols at the cathodic side. (b) Cathodic mass concentration and crossover rates of N‐propanol in the FB‐BPM system using CuSn catalysts, tested with alkaline, neutral, and acidic anolytes. (c) Stability test of the FB‐BPM system with CuSn catalysts for COR, tested with alkaline, neutral, and acidic anolytes. The current density is 100 mA/cm^2^ for Figure 4b,c.

Overall, the elevated K⁺ and OH^−^ concentrations at the cathodic surface enhanced the selectivity toward alcohols in COR, while the continuous flow of K⁺ toward the cathode suppressed liquid product crossover due to electroosmotic effects (Figure 4a). However, prolonged operation under high K⁺ and OH^−^ conditions may lead to instability. To address this, we replaced the strongly alkaline anolyte with neutral and acidic anolytes for COR testing in the FB‐BPM system (Figure 4b–c). The N‐propanol crossover rates remained stable at ∼10% with alkaline anolytes and at ∼12% with neutral anolytes. While the acidic anolyte and modified cation groups on the cathodic surface stabilized the cell voltage, the cathodic N‐propanol concentration remained relatively low (∼1 wt%). In contrast, the neutral anolyte led to a slightly lower cathodic N‐propanol concentration (∼6 wt%) but maintained a stable cell voltage for at least 60 h.

## Conclusions

This work investigated the COR performance of Cu alloy catalysts in a forward‐biased bipolar membrane MEA system. We found that the locally alkaline environment and the elevated K⁺ concentration enhanced multicarbon liquid product formation, while the FB‐BPM structure suppressed the liquid product crossover rate to <10%. The use in this system of CuSn catalysts enabled an alcohol stream concentration of > 15 wt% from the cathode gas channel.

## Conflict of Interests

The authors declare no conflict of interest.

## Supporting information



Supporting Information

## Data Availability

The data that support the findings of this study are available from the corresponding author upon reasonable request.

## References

[1] P. D. Luna , C. Hahn , D. Higgins , S. A. Jaffer , T. F. Jaramillo , E. H. Sargent , Science 2019, 364, 350. 10.1126/science.aav3506 31023896

[2] S. Nitopi , E. Bertheussen , S. B. Scott , X. Liu , A. K. Engstfeld , S. Horch , B. Seger , I. E. L. Stephens , K. Chan , C. Hahn , J. K. Nørskov , T. F. Jaramillo I. Chorkendorff . Chem. Rev. 2019, 119, 7610–7672, 10.1021/acs.chemrev.8b00705. 31117420

[3] H. Wu , L. Huang , J. Timoshenko , K. Qi , W. Wang , J. Liu , Y. Zhang , S. Yang , E. Petit , V. Flaud , J. Li , C. Salameh , P. Miele , L. Lajaunie , B. R. Cuenya , D. Rao , D. Voiry , Nat. Energy 2024, 9, 422–433, 10.1038/s41560-024-01461-6.

[4] S. Overa , B. S. Crandall , B. Shrimant , D. Tian , B. H. Ko , H. Shin , C. Bae , F. Jiao , Nat. Catal. 2022, 5, 738–745, 10.1038/s41929-022-00828-w.

[5] M. Jouny , W. Luc , F. Jiao , Nat. Catal. 2018, 1, 748–755, 10.1038/s41929-018-0133-2.

[6] F. P. G. D. Arquer , C. Dinh , A. Ozden , J. Wicks , C. McCallum , A. R. Kirmani , D. Nam , C. Gabardo , A. Seifitokaldani , X. Wang , Y. C. Li , F. Li , J. Edwards , L. J. Richter , S. J. Thorpe , D. Sinton , E. H. Sargent , Science 2020, 367, 661–666. 32029623 10.1126/science.aay4217 PMC13531662

[7] W. Ma , S. Xie , T. Liu , Q. Fan , J. Ye , F. Sun , Z. Jiang , Q. Zhang , J. Cheng , Y. Wang , Nat. Catal. 2020, 3, 478–487, 10.1038/s41929-020-0450-0.

[8] C. W. Li , J. Ciston , M. W. Kanan . Nature 2014, 508, 504–507, 10.1038/nature13249. 24717429

[9] P. Mardle , S. Cassegrain , F. Habibzadeh , Z. Shi , S. Holdcroft . J. Phys. Chem. C 2021, 125, 25446–25454, 10.1021/acs.jpcc.1c08430.

[10] J. A. Rabinowitz , M. W. Kanan , Nat. Commun. 2020, 11, 5231, 10.1038/s41467-020-19135-8. 33067444 PMC7567821

[11] L. Wang , S. Nitopi , A. B. Wong , J. L. Snider , A. C. Nielander , C. G. Morales‐Guio , M. Orazov , D. C. Higgins , C. Hahn , T. F. Jaramillo , Nat. Catal. 2019, 2, 702–708, 10.1038/s41929-019-0301-z.

[12] D. P. Gurudayal , S. Malani , Y. Lum , S. Haussener , J. W. Ager , ACS Appl. Energy Mater. 2019, 2, 4551–4559.

[13] M. A. Blommaert , D. Aili , R. A. Tufa , Q. Li , W. A. Smith , D. A. Vermaas , ACS Energy Lett. 2021, 6, 2539–2548, 10.1021/acsenergylett.1c00618. 34277948 PMC8276271

[14] J. C. Bui , E. W. Lees , D. H. Marin , T. N. Stovall , L. Chen , A. Kusoglu , A. C. Nielander , T. F. Jaramillo , S. W. Boettcher , A. T. Bell , A. Z. Weber , Nat. Chem. Eng. 2024, 1, 45–60, 10.1038/s44286-023-00009-x.

[15] P. Papangelakis , C. P. O'Brien , A. S. Zeraati , S. Liu , A. Paik , V. Nelson , S. Park , Y. C. Xiao , R. Dorakhan , P. Sun , J. Wu , C. M. Gabardo , N. Wang , R. K. Miao , E. H. Sargent , D. Sinton . Nat. Commun. 2025, 16, 4969. 40436848 10.1038/s41467-025-59180-9 PMC12120069

[16] H. P. Duong , J. G. R. de la Cruz , D. Portehault , A. Zitolo , J. Louis , S. Zanna , Q. Arnoux , M. W. Schreiber , N. Menguy , N. Tran , M. Fontecave , Nat. Mater. 2025, 24, 900–906, 10.1038/s41563-025-02153-6. 40074882

[17] Y. Chen , X. Wang , X. Li , R. K. Miao , J. Dong , Z. Zhao , C. Liu , J. E. Huang , J. Wu , S. Chu , W. Ni , Z. Guo , Y. Xu , P. Ou , B. Xu , Y. Hou , D. Sinton , E. H. Sargent , Nat. Catal. 2025, 8, 239–247, 10.1038/s41929-025-01301-0.

[18] S. Garg , C. A. G. Rodriguez , T. E. Rufford , J. R. Varcoe , B. Seger , Energy Environ. Sci. 2022, 15, 4440–4469, 10.1039/D2EE01818G.

[19] Z. Zhang , T. Wang , Y. Cai , X. Li , J. Ye , Y. Zhou , N. Tian , Z. Zhou , S. Sun , Nat. Catal. 2024, 7, 807–817, 10.1038/s41929-024-01179-4.

[20] Y. Xu , Z. Xia , W. Gao , H. Xiao , B. Xu , Nat. Catal. 2024, 7, 1120–1129, 10.1038/s41929-024-01227-z.

[21] X. Wang , Y. Chen , F. Li , R. K. Miao , J. E. Huang , Z. Zhao , X. Li , R. Dorakhan , S. Chu , J. Wu , S. Zheng , W. Ni , D. Kim , S. Park , Y. Liang , A. Ozden , P. Ou , Y. Hou , D. Sinton , E. H. Sargent , Nat. Commun. 2024, 15, 616, 10.1038/s41467-024-44727-z. 38242870 PMC10798983

[22] J. Park , Y. Ko , C. Lim , H. Kim , B. K. Min , K. Lee , J. H. Koh , H. Oh , W. H. Lee , Chem. Eng. J. 2023 453, 139826, 10.1016/j.cej.2022.139826.

[23] M. A. Blommaert , D. Alili , R. A. Tufa , Q. Li , W. A. Smith , D. A. Vermaas , ACS Energy Lett. 2021, 6, 2539–2548, 10.1021/acsenergylett.1c00618. 34277948 PMC8276271

[24] K. V. Petrov , C. I. Koopman , S. Subramanian , M. T. M. Koper , T. Burdyny , D. A. Vermaas . Nat. Energy 2024, 9, 932–938, 10.1038/s41560-024-01574-y.

[25] V. Okatenko , A. Loiudice , M. A. Newton , D. C. Stoian , A. Blokhina , A. N. Chen , K. Rossi , R. Buonsanti , J. Am. Chem. Soc. 2023, 145, 5370–5383, 10.1021/jacs.2c13437. 36847799

[26] X. Chang , Y. Zhao , B. Xu , ACS Catal. 2020, 10, 13737–13747, 10.1021/acscatal.0c03108.

[27] J. Zhang , C. Zhang , M. Wang , Y. Mao , B. Wu , Q. Yang , B. Wang , Z. Mi , M. Zhang , N. Ling , W. R. Leow , Z. Wang , Y. Lum , Nat. Chem. 2025, 17, 334–343, 10.1038/s41557-024-01721-8. 39915658

[28] H. Peng , M. T. Tang , J. H. Stenlid , X. Liu , F. Abild‐Pedersen , Nat. Commun. 2022, 13, 1399, 10.1038/s41467-022-29140-8. 35302055 PMC8931056

[29] M. Luo , Z. Wang , Y. C. Li , J. Li , F. Li , Y. Lum , D. Nam , B. Chen , J. Wicks , A. Xu , T. Zhuang , W. R. Leow , X. Wang , C. Dinh , Y. Wang , Y. Wang , D. Sinton , E. H. Sargent , Nat. Commun. 2019, 10, 5814, 10.1038/s41467-019-13833-8. 31862886 PMC6925210

[30] K. P. Kepp , Inorg. Chem. 2016, 55, 9461–9470. 27580183 10.1021/acs.inorgchem.6b01702

[31] Y. Ji , Z. Chen , R. Wei , C. Yang , Y. Wang , J. Xu , H. Zhang , A. Guan , J. Chen , T. Sham , J. Luo , Y. Yang , X. Xu , G. Zheng , Nat. Catal. 2022, 5, 251–258 10.1038/s41929-022-00757-8.

[32] H. Shen , Y. Wang , T. Chakraborty , G. Zhou , C. Wang , X. Fu , Y. Wang , J. Zhang , C. Li , F. Xu , L. Cao , T. Mueller , C. Wang . ACS Catal. 2022, 12, 5275−5283 10.1021/acscatal.2c00646.

[33] J. Li , H. Xiong , X. Liu , D. Wu , D. Su , B. Xu , Q. Lu , Nat. Commun. 2023, 14, 698, 10.1038/s41467-023-36411-5 36755022 PMC9908878

[34] J. Jin , J. Wicks , Q. Min , J. Li , Y. Hu , J. Ma , Y. Wang , Z. Jiang , Y. Xu , R. Lu , G. Si , P. Papangelakis , M. Shakouri , Q. Xiao , P. Ou , X. Wang , Z. Chen , W. Zhang , K. Yu , J. Song , X. Jiang , P. Qiu , Y. Lou , D. Wu , Y. Mao , A. Ozden , C. Wang , B. Y. Xia , X. Hu , V. P. Dravid , Y. Yiu , T. Sham , Z. Wang , D. Sinton , L. Mai , E. H. Sargent , Y. Pang . Nature 2023, 617, 724–729. 37138081 10.1038/s41586-023-05918-8

[35] X. Wang , Y. Chen , F. Li , R. K. Miao , J. E. Huang , Z. Zhao , X. Li , R. Dorakhan , S. Chu , J. Wu , S. Zheng , W. Ni , D. Kim , S. Park , Y. Liang , A. Ozden , P. Ou , Y. Hou , D. Sinton , E. H. Sargent , Nat. Commun. 2024, 15, 616, 10.1038/s41467-024-44727-z. 38242870 PMC10798983

