# Supplementary material for: High‐Concentration Alcohol Generation in Bipolar Membrane CO Electrolyzer
Source: Angew Chem Int Ed Engl. 2025 Nov 18;65(3):e17470. doi: 10.1002/anie.202517470 (PMC12811668; doi:10.1002/anie.202517470)
Supplement: Supplementary file 1 — Supporting Information [file ANIE-65-e17470-s001.docx]

Supplementary Information

High-Concentration Alcohol Generation in Bipolar Membrane CO Electrolyzer

Wenjin Zhu^1^†, Qiu-Cheng Chen^1^†, Yiqing Chen^1^†, Jianan Erick Huang^1^†, Guangcan Su^1^, Hengzhou Liu^1^, Weiyan Ni^1^, Yuanjun Chen^1^, Jiaqi Yu^1^, Bosi Peng^1^, Jiantao Li^1^, Sungsik Lee^2^, Rong Xia^1^, Shaoyun Hao^1^, Yuxia Shen^1^, Huajie Ze^1^, Bei Zhou^1^, Xiao-Yan Li^1^, Yali Ji^1^, Shuang Yang^1^, Cong Tian^1^, Yongxiang Liang^1^, Ke Xie^1^*, Edward H. Sargent^1^*

[1] Dr. W. Zhu†, Dr. Q. Chen†, Dr. Y. Chen†, Dr. J. E. Huang†, Dr. G. Su, Dr. H. Liu, Dr. W. Ni, Dr. Y. Chen, Dr. J. Yu, Dr. B. Peng, Dr. J. Li, Dr. R. Xia, Dr. S. Hao, Dr. Y. Shen, Dr. H. Ze, Dr. B. Zhou, Dr. X. Li, Dr. Y. Ji, Dr. S. Yang, Dr. C. Tian, Dr. Y. Liang, Dr. K. Xie, Prof. E. H. Sargent

Department of Chemistry and Department of Electrical and Computer Engineering, Northwestern University, Evanston, Illinois, USA

E-mail: ke-xie@northwestern.edu, ted.sargent@northwestern.edu

[2] Dr. S. Lee
X-ray Science Division, Argonne National Laboratory, Lemont, Illinois, USA

[†] These authors contributed equally to the work

**Contents**

Supplementary 1. Materials and Methods………………………………………………………………….………3

Supplementary 2. Characterizations of Cu Catalysts……………………………………………………………..5

Supplementary 3. Baseline COR Tests of Cu Catalyst in MEA Configuration…………………………………8

Supplementary 4. Understandings of Liquid Product Crossover in MEA Systems…………………………...12

Supplementary 5. Comparisons of CEM and RB-BPM for Cu-catalyzed COR………………………………14

Supplementary 6. Investigations of Cu-Catalyzed COR in FB-BPM System…………………………………18

Supplementary 7. Energy Cost Analysis for Acetate vs. Alcohol Production…………………………………21

Supplementary 8. Computational Screening of Catalysts for Enhanced Alcohol Selectivity in COR………22

Supplementary 9. Experimental Screening of Catalysts for Enhanced Alcohol Selectivity in COR…………26

Supplementary 10. Materials Characterization and COR Performance of CuZn Catalyst in K^+^-FB-BPM System……………………………………………………………………………………………………………….29

Supplementary 11. Materials Characterization and COR Performance of CuSn Catalyst in K^+^-FB-BPM System…....................................................................................................................................................36

Supplementary 12. Investigations on the Concentration of K^+^ and OH^-^ for CuSn Catalyst in K^+^-FB-BPM System……………………………………………………………………………………………………………….40

References…………………………………………………………………………………………………………..41

**Supplementary 1. Materials and Methods**

**Chemicals and Materials**

Copper(II) nitrate trihydrate, zinc sulfate heptahydrate, tungsten chloride, titanium butoxide, manganese chloride tetrahydrate, ruthenium chloride trihydrate, iron nitrate nonahydrate, cobalt acetate tetrahydrate, potassium hydroxide, and graphene oxide were purchased from Sigma-Aldrich. Tin(IV) chloride, selenium tetrachloride, antimony(III) chloride, tellurium(IV) chloride, nickel(II) chloride hexahydrate, and potassium hydrocarbonate were purchased from Thermo Scientific. Silver nitrate, palladium(II) chloride, and dimethyl sulfoxide-d6 were purchased from Fisher Scientific. Lead chloride was purchased from Tokyo Chemical Industry Co., Ltd. Freudenberg H23C3 and nickel foam were purchased from Fuel Cell Store. Sustainion® X37-50 Grade RT membrane and Nafion™ NR212 membrane were purchased from Dioxide Materials. Gases (air, hydrogen, argon) were purchased from Airgas, methanol from MilliporeSigma, and deuterium oxide from Cambridge Isotope Laboratories. Deionized water (18.2 MΩ) was used for electrode preparation. All chemicals were used as received without further purification.

**Catalysts Synthesis**

The Cu-based catalyst was synthesized via a hydrothermal method, similar with a previously reported procedure^[1]^. 1 g of copper(II) nitrate trihydrate was dissolved in 50 mL deionized water under vigorous magnetic stirring in an ice-water bath. Then, 10 mL of 1 M NaOH was added dropwise, and stirring continued for 30 minutes. The mixture was stored at 3°C for 24 h before being transferred to a Teflon-lined autoclave and heated at 130°C for 18 h. After cooling to room temperature, the catalyst was collected by centrifugation, washed twice with deionized water, and dispersed in 20 mL methanol for storage.

For other bimetallic catalysts, a similar procedure was followed, with the additive metal (1 wt% relative to Cu) incorporated during synthesis.

**Electrode Preparation**

The catalyst ink was prepared by mixing 150 μL of Nafion perfluorinated resin solution with 6 mL of a homogeneous methanol suspension containing the catalyst, followed by sonication in a glass vial. The resulting ink was spray-coated onto a gas diffusion layer (Freudenberg H23C3) to achieve a catalyst loading of ~2 mg/cm².

**Material Characterization**

Scanning electron microscopy (SEM) was performed using a Hitachi SU8030 field-emission microscope. High-resolution TEM (HRTEM), HAADF-STEM, and elemental mapping were conducted on a JEOL 2100F microscope (200 kV) equipped with a probe spherical aberration corrector. X-ray photoelectron spectroscopy (XPS) was carried out on a Thermo Scientific NEXSA G2 system with an Al Kα X-ray source. In situ Raman spectroscopy was performed using a Renishaw inVia microscope with a ×63 water immersion objective and a 785 nm laser in a custom flow cell. Metal composition was determined by energy-dispersive X-ray fluorescence (ED-XRF, Xenemetrix). X-ray absorption spectroscopy (XAS) measurements were conducted at Beamline 12-BM of the Advanced Photon Source (APS, Argonne National Laboratory), with data processed using Athena and Artemis software.

**Electrochemical Measurements**

CO₂ reduction reaction (CORR) tests were performed in a membrane electrode assembly (MEA) setup at room temperature using an ECLAB VMP3B-5 potentiostat. Nickel foam served as the anode. Unless explicitly stated otherwise, 1 M KOH was continuously fed into the anode chamber at 20 mL·min⁻¹ via a peristaltic pump. CO was supplied to the cathode at 30 sccm using a digital mass flow controller. The compact MEA design minimized interelectrode distance, reducing ohmic losses.

**Product analysis**

Gas-phase products were collected from the MEA cell outlet using gas-tight syringes (Hamilton) and analyzed via offline gas chromatography (Agilent Nexis GC-2030). The gas chromatograph (GC) system was equipped with: a flame ionization detector (FID) for CH₄ and C₂H₄ quantification; a thermal conductivity detector (TCD) for H₂, O₂, N₂, and CO detection. For each measurement, 2 mL of gas was injected into the GC. Liquid products were analyzed by ¹H NMR spectroscopy (600 MHz Agilent DD2) in water suppression mode, using dimethyl sulfoxide (DMSO) as an internal standard and deuterium oxide (D₂O) as the lock solvent. Faradaic efficiency (FE) was calculated for both anode and cathode products using:

$$FE=\frac{N\times F\times n}{Q}$$

Where $N=$ electrons transferred per molecule, $F$ = Faraday constant (96,485 C/mol), $n$= total moles of products, $Q$ = total charge passed during electrolysis.

**Supplementary 2. Characterizations of Cu Catalysts**


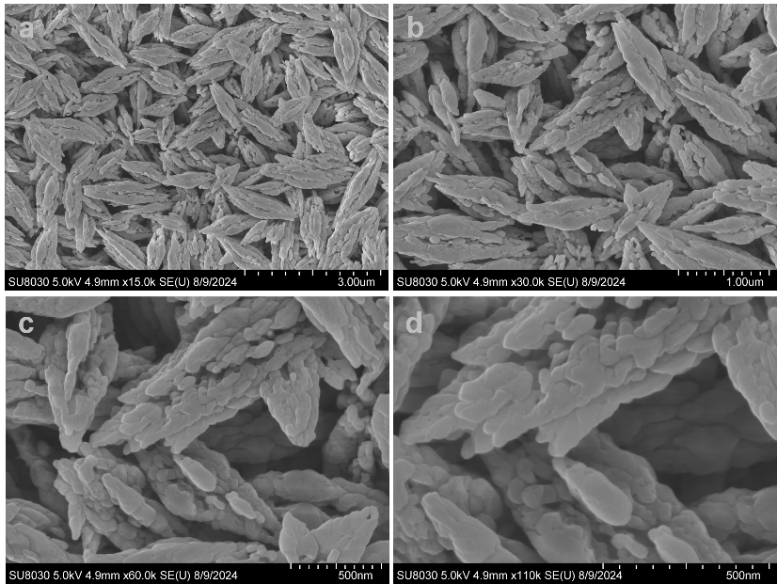


**Supplementary Fig. 1 SEM images of the Cu catalyst deposited on carbon paper before electrochemical reaction.** SEM images revealing a dendritic morphology with particle sizes ranging from hundreds of nanometers to several micrometers.


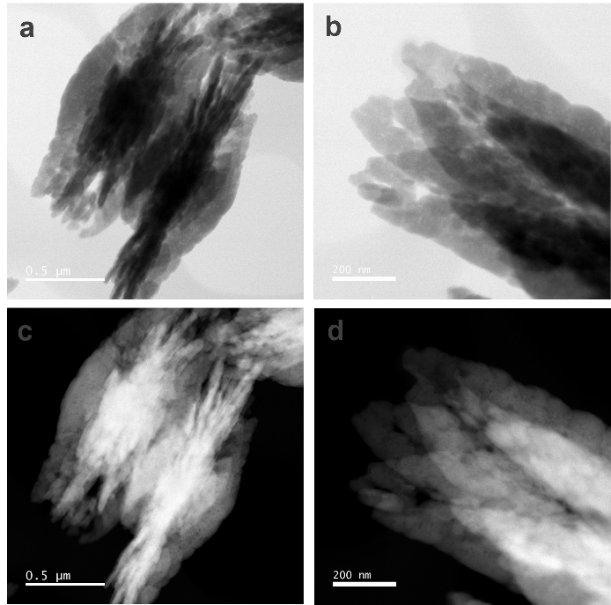


**Supplementary Fig. 2 TEM images of the Cu catalyst deposited on carbon paper before electrochemical reaction.** (a-b) Low resolution TEM images of Cu catalyst, (c-d) HAADF STEM images of Cu catalyst.

**Supplementary Fig. 3** **XPS spectra of the Cu catalyst before electrochemical reaction**. The Cu 2p₃/₂ peak at 933.6 eV, along with its satellite feature, confirms the presence of CuOx rather than metallic Cu.

**Supplementary 3. Baseline COR Tests of Cu Catalyst in MEA Configuration**


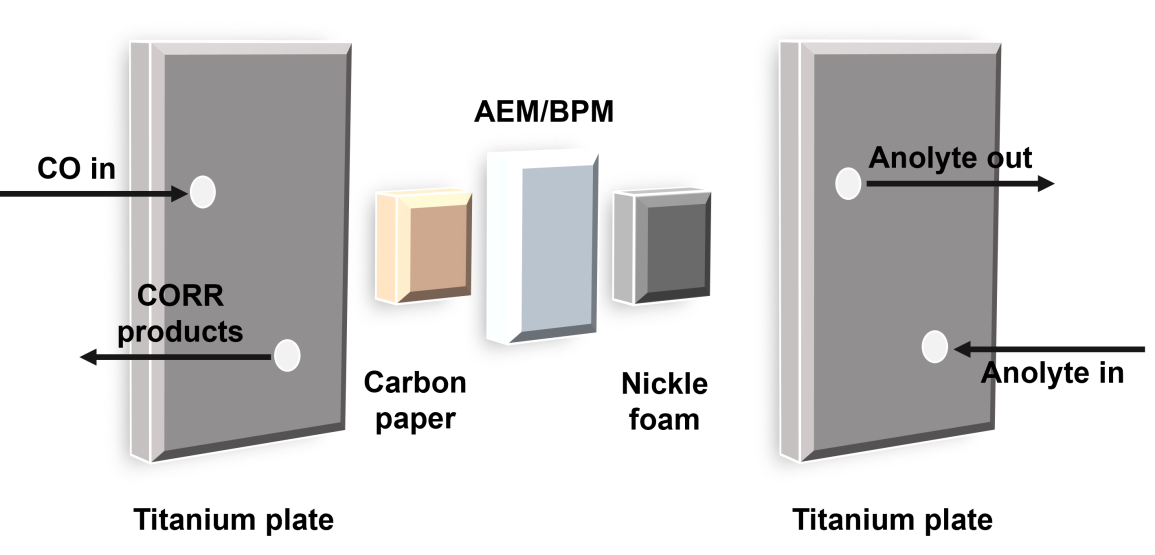


**Supplementary Fig. 4 Schematic of the MEA setup.** Components: Two titanium plates, carbon paper-supported cathode, nickel foam anode, and either AEM or BPM for ion exchange.

**
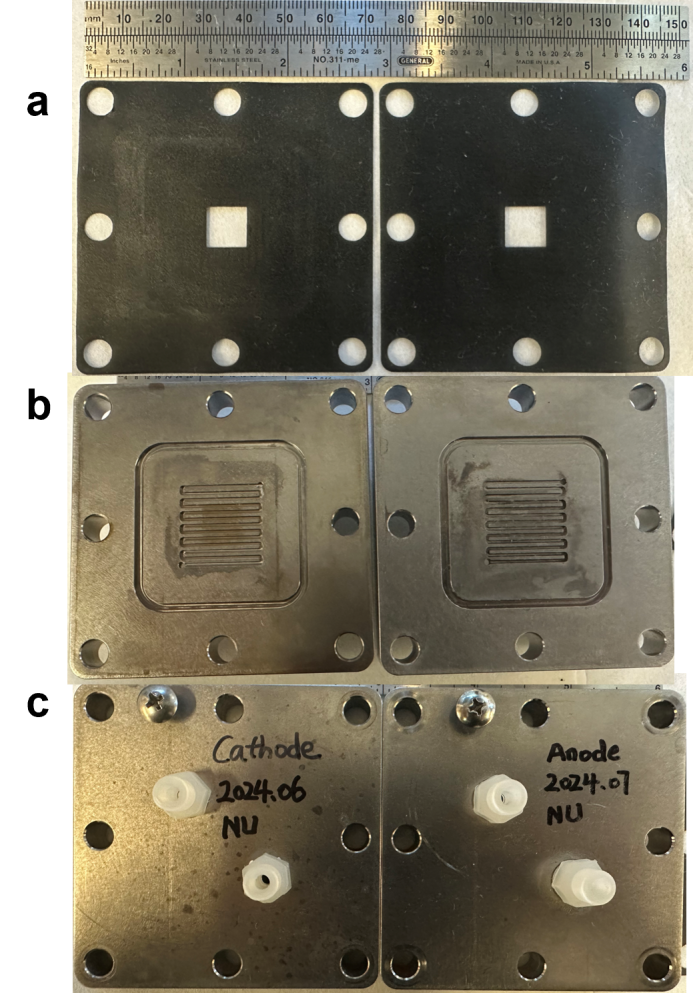
**

**Supplementary Fig. 5 MEA assembly details.** (a) Silicone gaskets (1 cm × 1 cm window); (b) Inner titanium plate (2.2 cm × 2.2 cm flow channels); (c) Outer titanium plate.


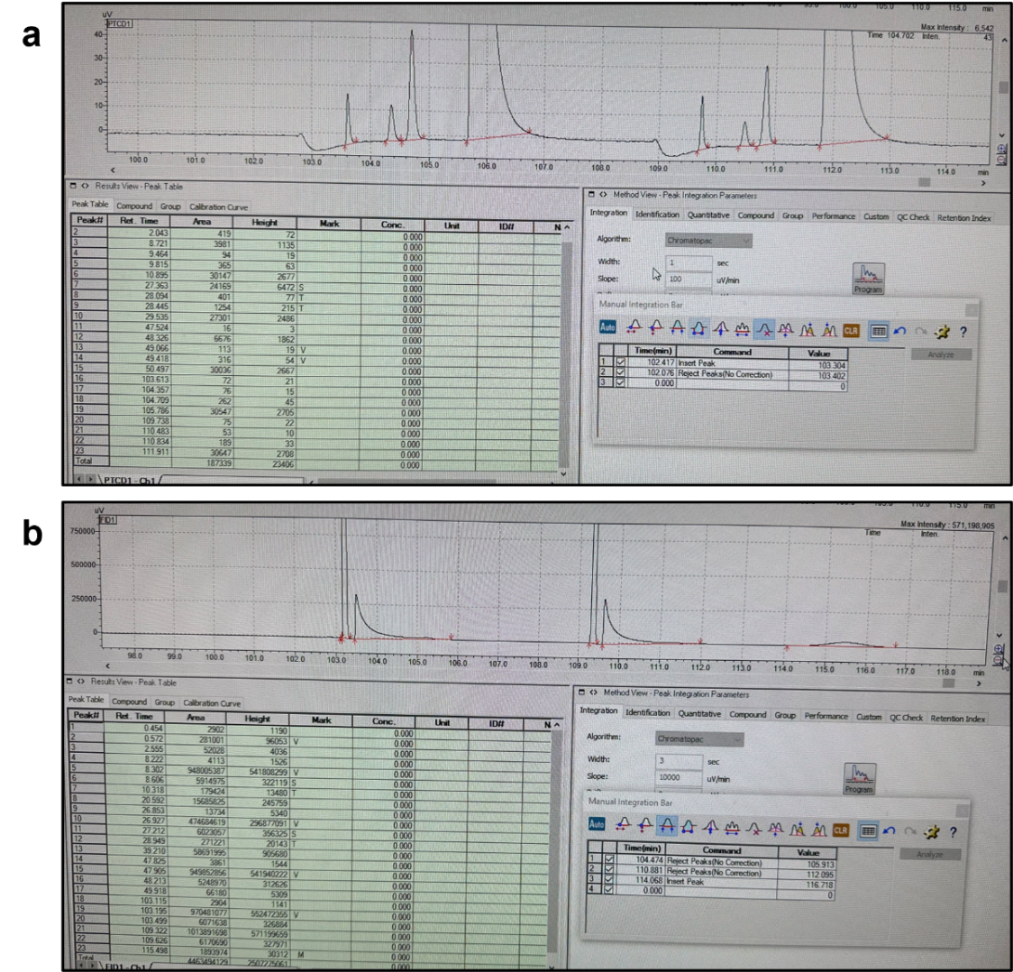


**Supplementary Fig. 6** **Representative gas chromatography analysis of COR products**. (a) TCD spectrum: Peaks for H₂ and CO (negligible O₂/N₂ confirms system airtightness). (b) FID spectrum: C₂H₄ detection.


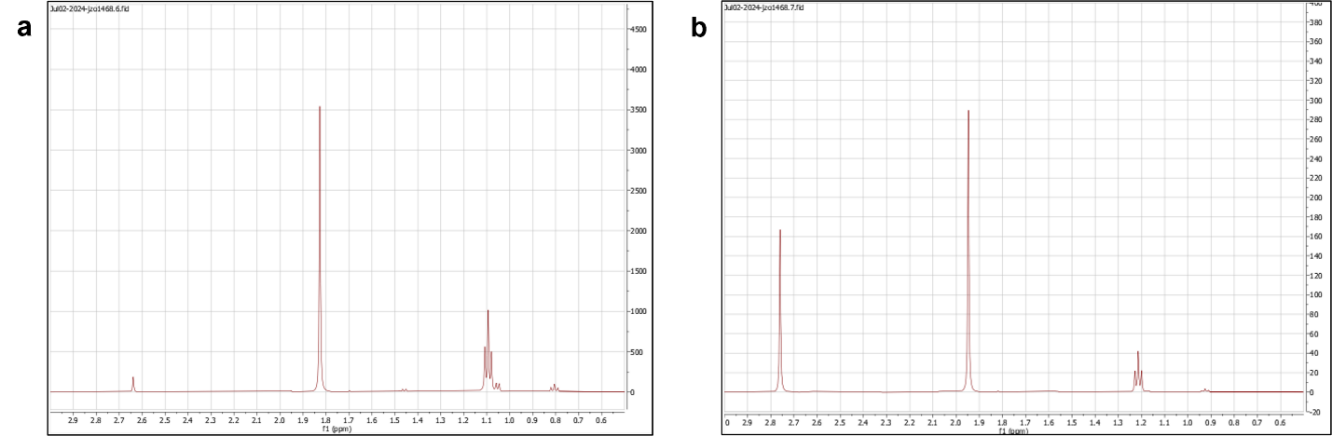


**Supplementary Fig. 7 Representative ¹H-NMR analysis of liquid products (DMSO internal standard, δ = 2.7 ppm).** Representative ^1^H-NMR spectrum with Cu catalyst at -100 mA/cm^2^ , 1 M KOH aqueous solution as the anolyte. Liquid products include CH_3_COO^-^, EtOH, n-PrOH. (a) the liquid collected from cathode, (b) the liquid collected from anode.

**Supplementary 4. Understandings of Liquid Product Crossover in MEA Systems**

**Supplementary Fig. 8** **Ethanol Crossover Under Concentration Gradients.** To investigate the alcohol crossover in the absence of applied bias by looking solely at the passive membrane itself in the presence of a concentration gradient, we introduced pure ethanol of 2 M on the cathode, pure water on the anode, and circulated the solution for 60 minutes the case of each membrane, and measured the quantity of ethanol on the anode. The 50 um AEM had the highest loss due to ethanol crossover, with ~5% lost to the anode; the CEMs had ~4% loss; and the 50um:50 um AEL:CEL FB-BPM had ~1% crossover. These results demonstrate that the FB-BPM provides the highest resistance to concentration-driven ethanol diffusion, although the differences between membranes were relatively small.

**Supplementary Fig. 9** **Ethanol crossover in water splitting system.** To evaluate electro-osmotic drag effects, we circulated 1 M ethanol aqueous solution at the cathode while maintaining pure water at the anode. After 60 minutes, the 50 μm AEM showed significant ethanol crossover (~25% to anode), while the 50 μm:50 μm AEL:CEL FB-BPM demonstrated minimal crossover (~3%). These results highlight the critical influence of electro-osmotic forces on product migration across membranes.

**Supplementary 5. Comparisons of CEM and RB-BPM for Cu-catalyzed COR**


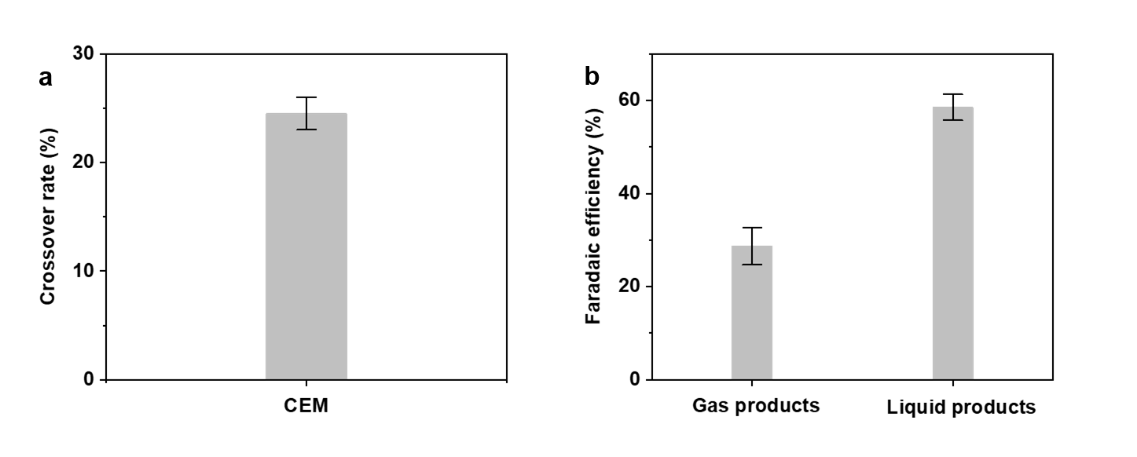


**Supplementary Fig. 10** **COR performance of Cu catalyst with CEM** **at -100 mA/cm^2^ with 1 M KOH anolyte.** (a) The crossover rate and (b) Products distribution for Cu catalyst towards COR.


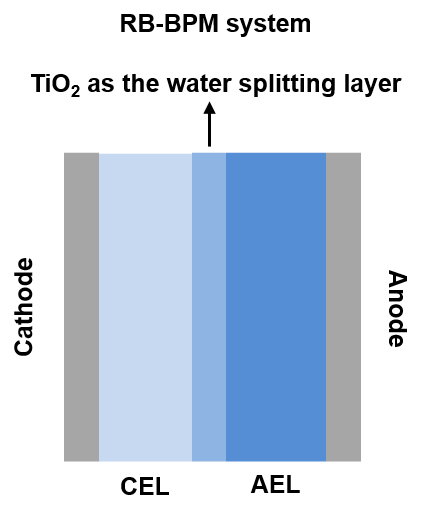


**Supplementary Fig. 11** **Schematic illustration of the RB-BPM system configuration.**


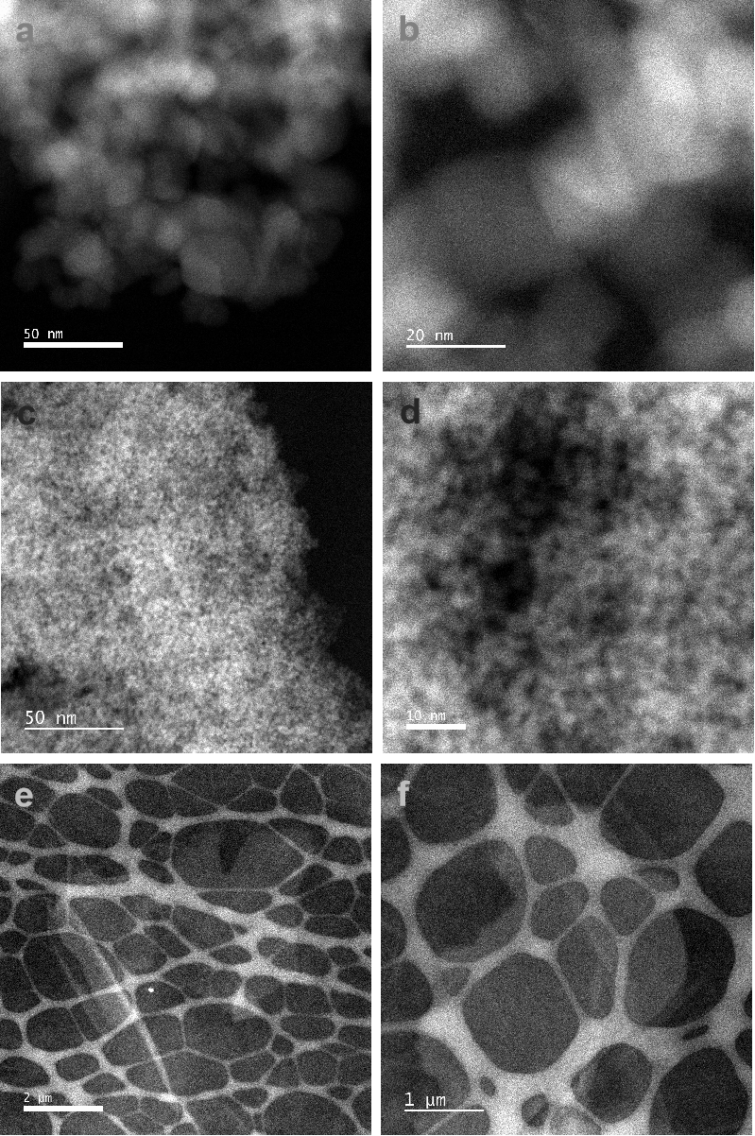


**Supplementary Fig. 12** **Aberration-corrected high angle annular dark-field scanning transmission electron microscopy (AC HAADF-STEM) images of water dissociation catalysts** **employed in the RB-BPM system.** (a-b) TiO_2_ nanoparticles, with the size <50 nm.

**Supplementary Fig. 13** **Cell voltage of Cu catalyst with RB-BPM for COR in MEA configuration at -100 mA/cm^2^ with 1 M KOH anolyte.**

**Supplementary 6. Investigation of Cu-Catalyzed COR in FB-BPM System**


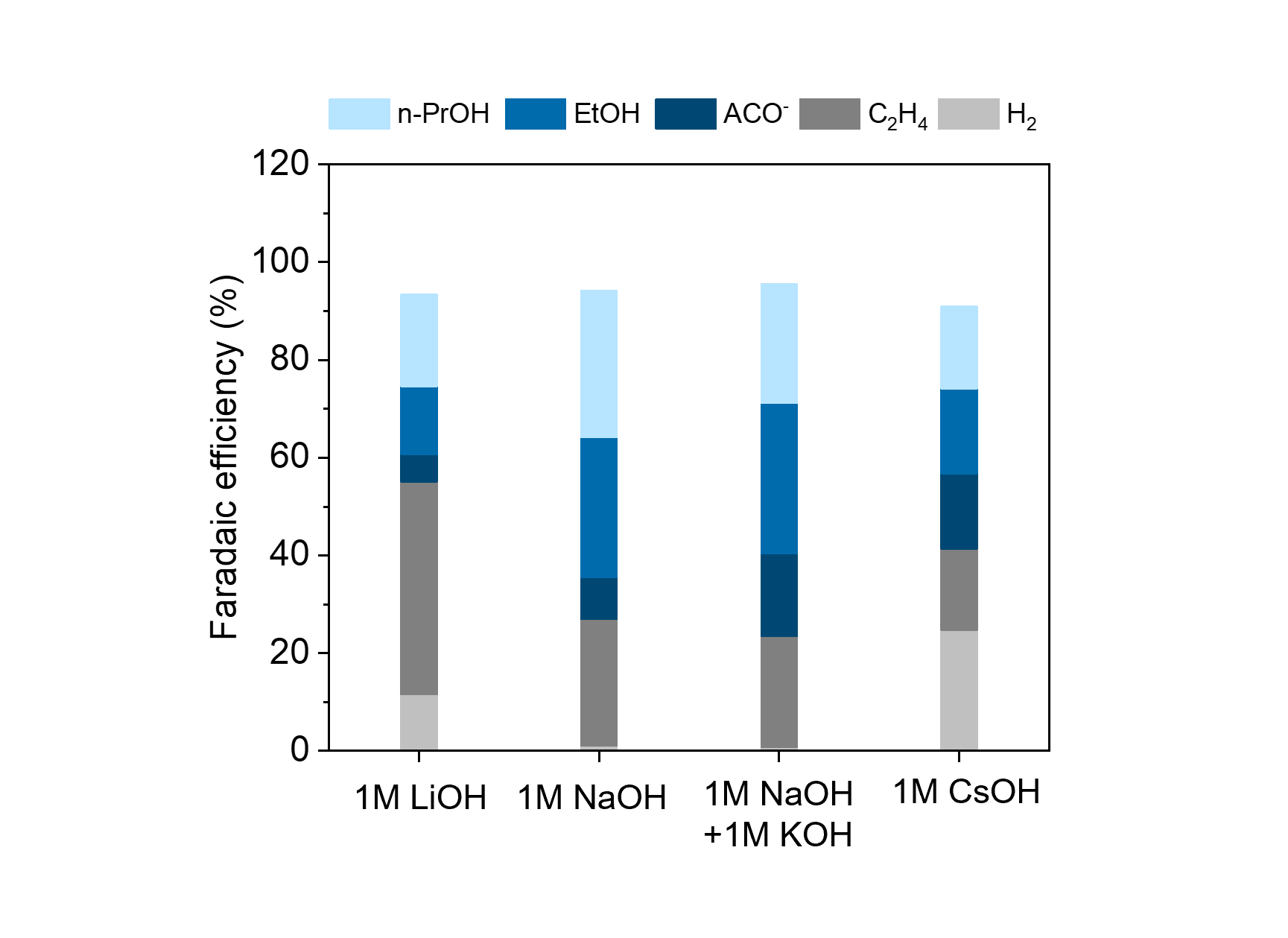


**Supplementary Fig. 14 COR performance of Cu catalyst at -100 mA/cm^2^ with different anolytes.** Alkaline anolytes: 1 M LiOH, 1 M NaOH, 1 M NaOH + 1 M KOH, and 1 M CsOH. These system demonstrated higher Faradaic efficiency for gaseous products compared to 1 M KOH alone. These results highlight the critical role of K⁺ ions in promoting multicarbon liquid product formation during COR.


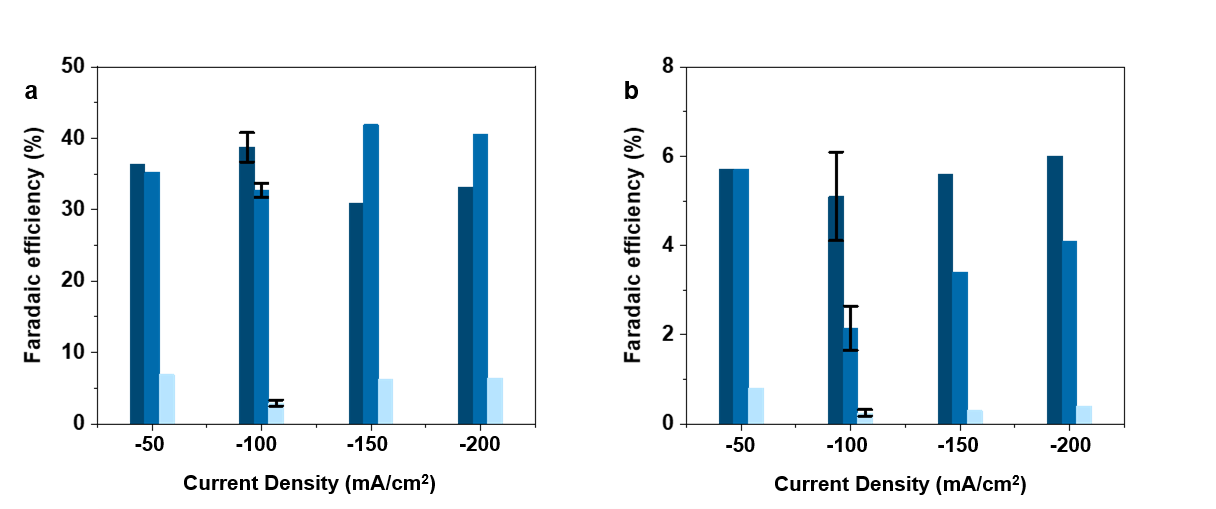


**Supplementary Fig. 15 COR performance of Cu catalyst at different current densities in FB-BPM system.** (a) Faradaic efficiency of liquid products collected from the cathode. (b) Faradaic efficiency of liquid products collected from the anode. Test conditions: -100 mA/cm^2^, 1 M KOH anolyte.

**Supplementary Fig. 16 The mass concentration of alcohols from the cathodic outlet channel at -100 mA/cm^2^ in K^+^-FB-BPM system.** The mass percentage is 4.1 wt% for ethanol and 0.3 wt% for n-propanol. The alcohol mass concentration calculation was based on the 30-40 minutes of reaction interval.

**Supplementary 7. Energy Cost Analysis for Acetate vs. Alcohol Production**

**Supplementary Fig. 17 A comparison of the energy costs for purifying liquid products,** **using acetate and ethanol (representative of alcohols) as model systems.** The separation was simulated in Aspen Plus to quantify the energy input required for purification via distillation. Acetic acid was recovered through a combined solvent extraction and distillation process using the ELECNRTL thermodynamic model, which accounts for electrolyte interactions and non-ideal phase behavior. The acidified feed underwent phase separation, followed by selective extraction of acetic acid using ethyl acetate. Glacial acetic acid (>99.5 wt%) was then obtained via distillation, while the solvent was recovered through phase separation and recycled in a closed-loop configuration. Based on literature values, the energy requirement for KOH recovery from KCl was estimated at approximately 1.58 kWh/kg KOH ^[2]^. The total energy input to separate 1 tonne of acetic acid from a 50 wt% acetate solution was about 13.9 GJ. Ethanol purification was modeled using a two-column distillation configuration with the NRTL thermodynamic model. The first column concentrated ethanol in the overhead, which was subsequently dehydrated in a second column to achieve >99.5 wt% purity. The total energy input to separate 1 tonne of ethanol from a 50 wt% ethanol solution was approximately 7.8 GJ. The total energy cost for acetate purification was 1.8 times of that for ethanol. Given this significant difference, also alcohol is a much more energy dense chemical with larger market demand, we prioritize improving alcohols selectivity as our main objective in the following chapter.

**Supplementary 8.** **Computational Screening of Catalysts for Enhanced Alcohol Selectivity in COR**

Spin-polarized density functional theory (DFT) calculations^[3]^ were carried out using the Vienna Ab Initio Simulation Package (VASP)^[4]^. Electron-ion interactions were modeled via the projector-augmented wave (PAW) approach^[5]^, while exchange-correlation effects were treated within the generalized gradient approximation (GGA) using the Perdew-Burke-Ernzerhof (PBE) functional^[6]^. To account for long-range dispersion forces, Grimme's DFT-D3(BJ) scheme was applied^[7]^. An energy cutoff of 450 eV was employed. Structural optimizations were performed until atomic forces dropped below 0.02 eV/Å and energy changes were within 10^-5^ eV. The solvent environment was modeled using the implicit solvation approach implemented in VASPsol++^[8]^. A constant electrode potential of –0.4 V vs. SHE was applied to all slabs. The electrolyte concentration was set to 1 mol/L, and an ionic radius of 4 Å was employed for constructing the ionic cavity.

For the pristine Cu surface, a 3 × 3 Cu(111) supercell comprising four atomic layers was constructed, with the bottom two layers fixed at their bulk lattice positions. Metal-doped systems were generated by substituting a single surface Cu atom on Cu(111) with the dopant atom. Additionally, a vacuum layer of at least 15 Å was introduced perpendicular to the surface to minimize interactions between periodic images.


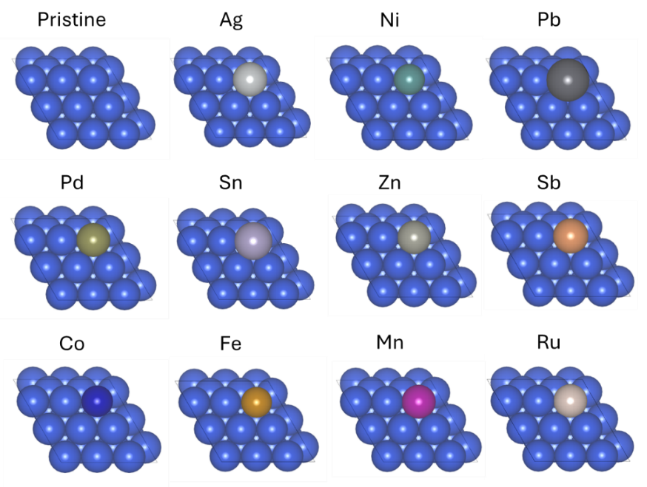


**Supplementary Fig. 18 Top views of the pristine Cu (111) and metal-doped Cu (111) surfaces.**


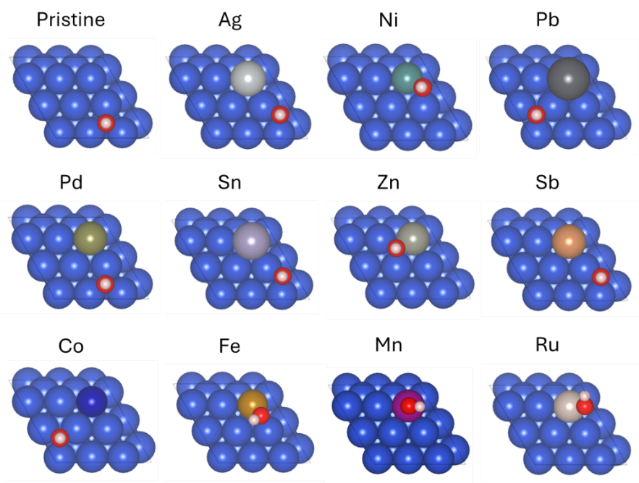


**Supplementary Fig. 19 Optimized geometries of *OH adsorbed on pristine Cu (111) and metal-doped Cu (111) surface.**


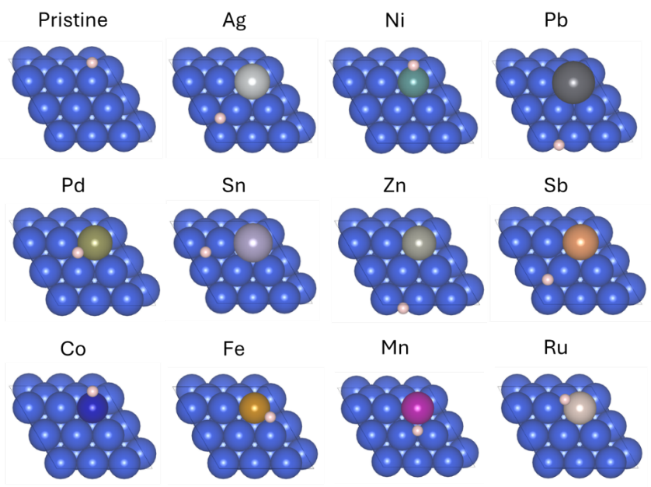


**Supplementary Fig. 20 Optimized geometries of *H adsorbed on pristine Cu (111) and metal-doped Cu (111) surface.**

**Supplementary 9. Experimental Catalyst Screening for Enhanced Alcohol Selectivity in COR**


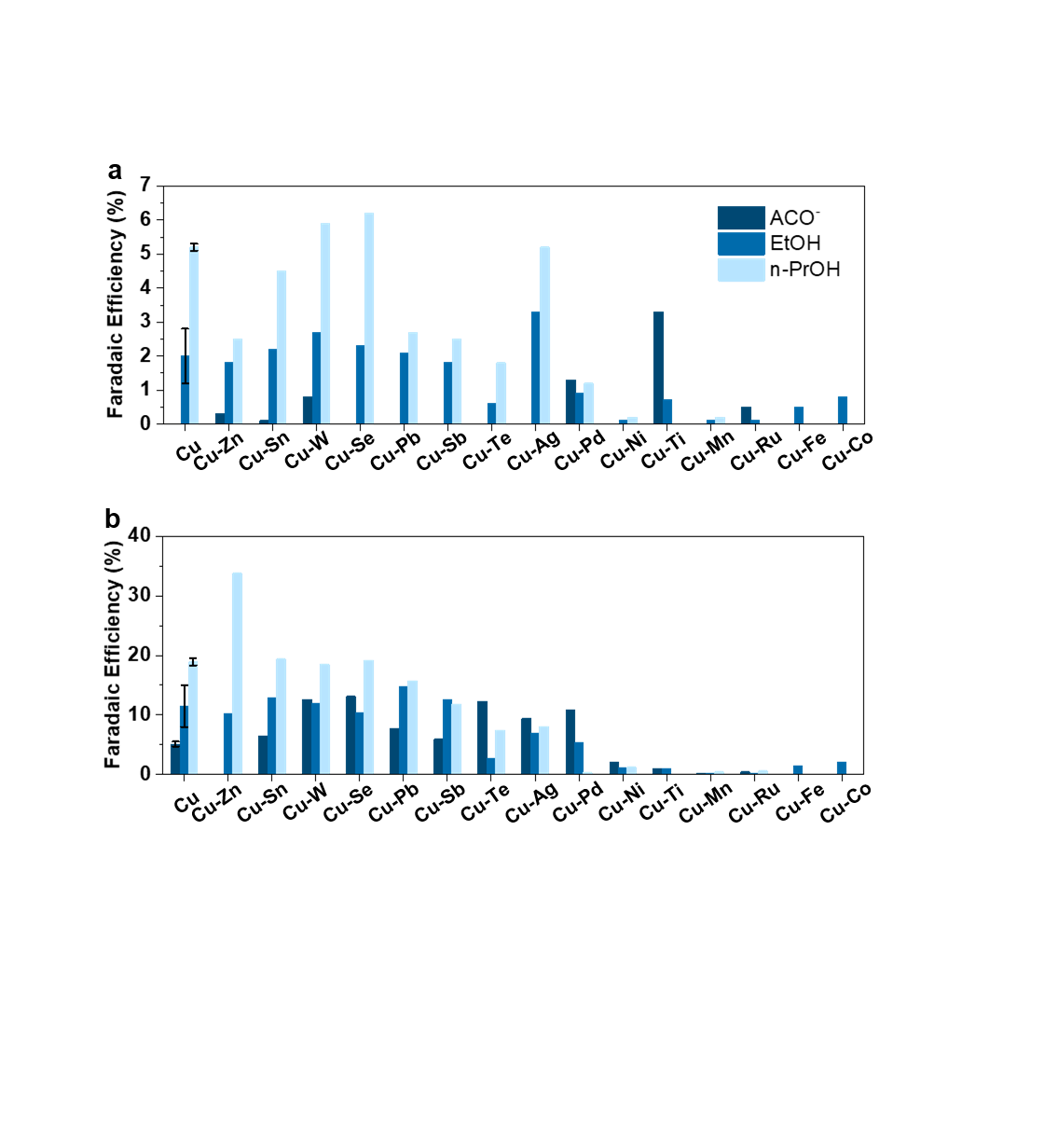


**Supplementary Fig. 21 COR performance of Cu-M bimetallic catalysts in K^+^-AEM system at -100 mA/cm^2^ with 1 M KOH anolyte.** (a) Faradaic efficiency of cathodic liquid products, (b) Faradaic efficiency of anodic liquid products.


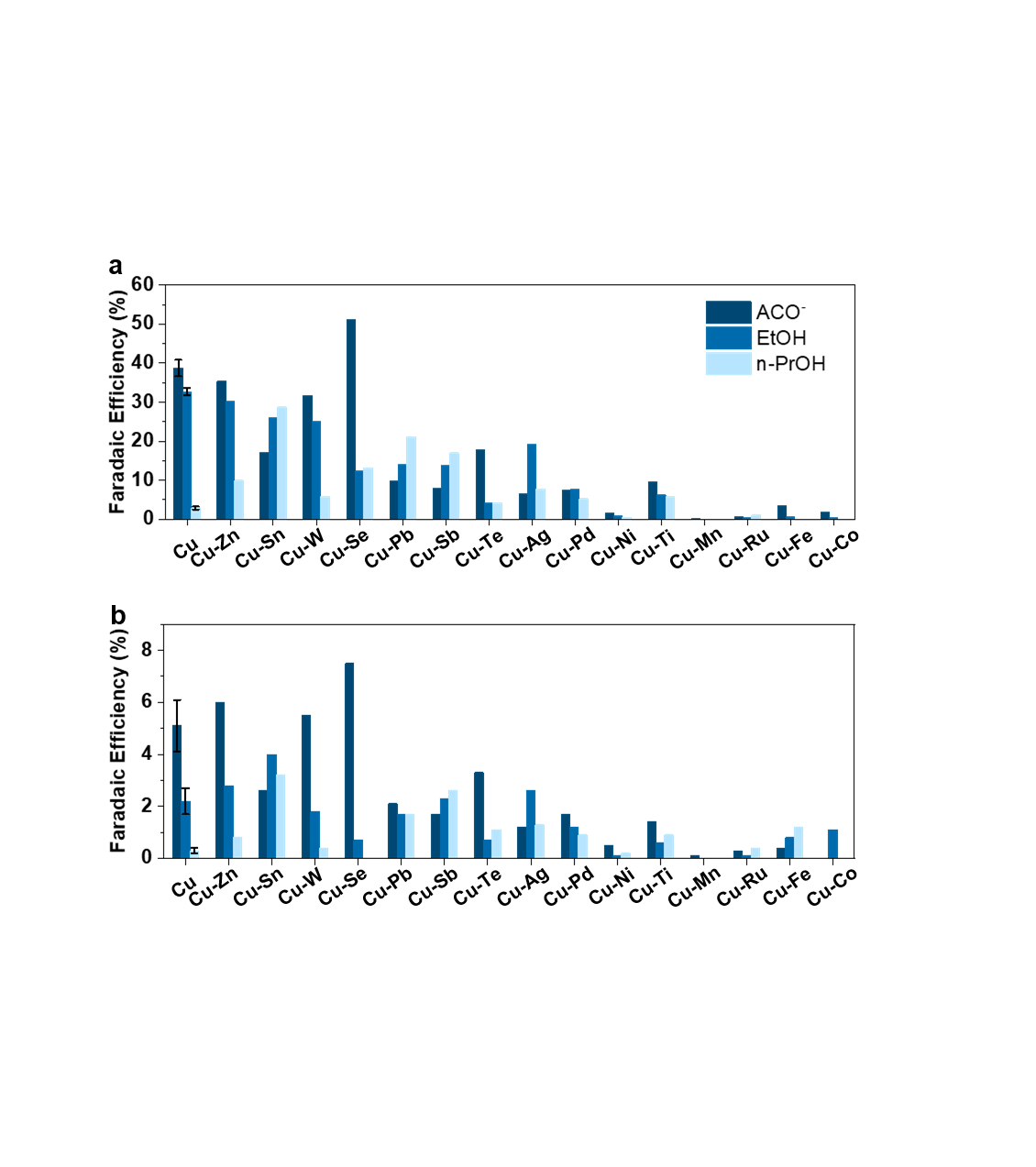


**Supplementary Fig. 22 COR performance of** **Cu-M bimetallic catalysts in K^+^-FB-BPM system at -100 mA/cm^2^ with 1 M KOH anolyte.** (a) Faradaic efficiency of cathodic liquid products, (b) Faradaic efficiency of anodic liquid products.


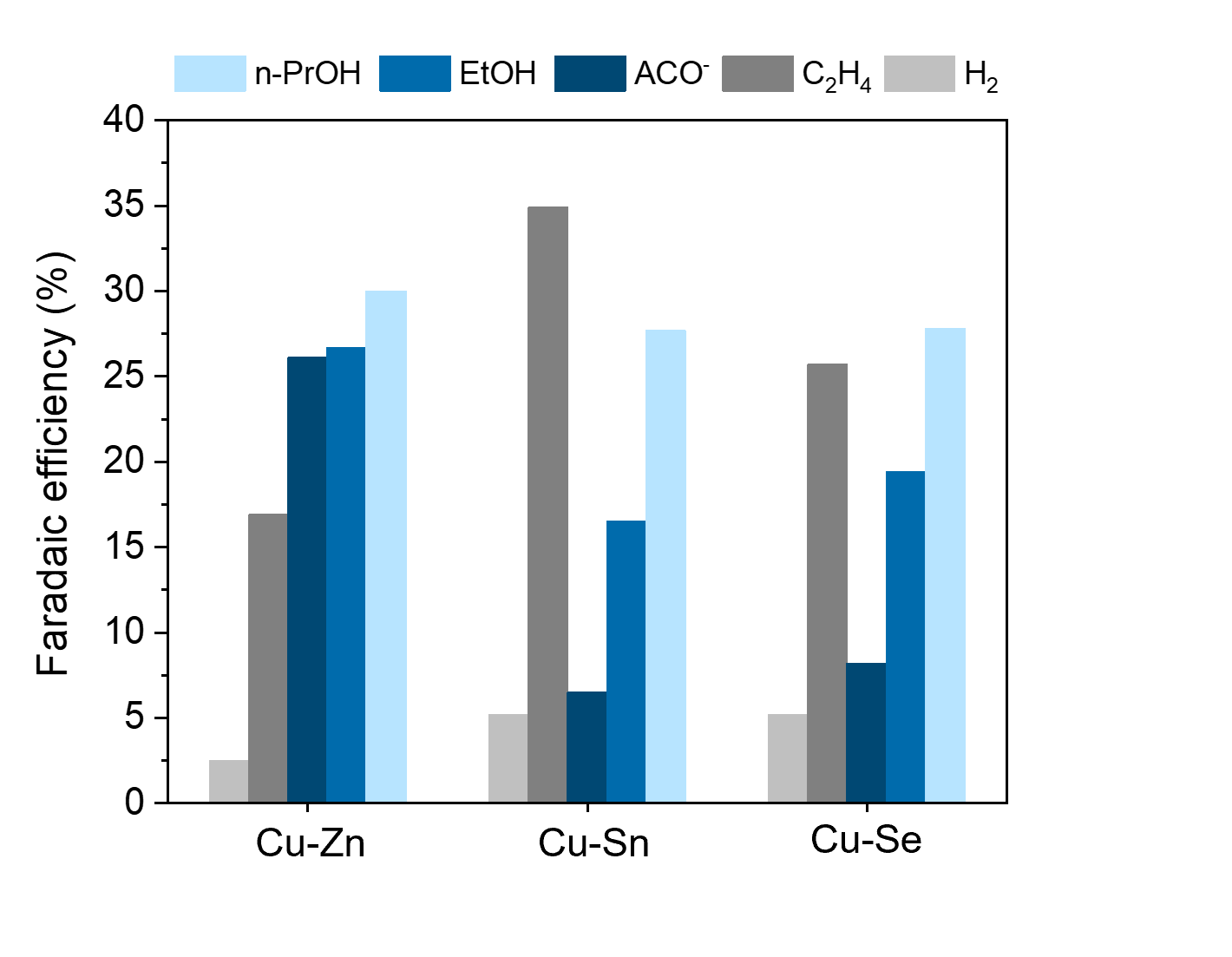


**Supplementary Fig. 23 Faradaic efficiency of Cu-M bimetallic catalysts in Na^+^-FB-BPM system at -100 mA/cm^2^ with 1 M NaOH anolyte.** Relative to Cu-M performance in 1 M KOH aqueous solution, the Na^+^-FB-BPM system exhibits increased C₂H₄ FE and decreased total liquid product FE. These results demonstrate the critical role of cations in modulating COR product distribution. Specifically, K⁺ in this FB-BPM system significantly suppresses the FE of gaseous products while promoting liquid products formation.

**Supplementary 10. Materials Characterization and COR Performance of CuZn Catalyst in K^+^-FB-BPM System**


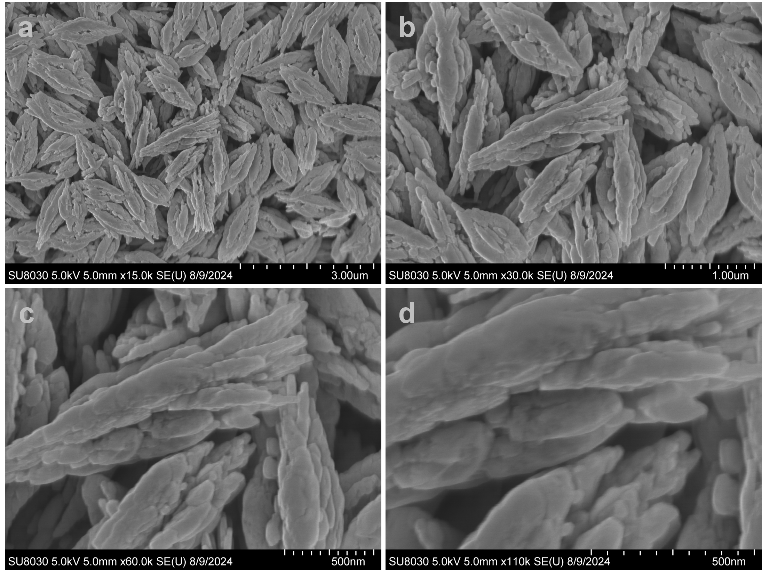


**Supplementary Fig. 24 SEM characterization of CuZn catalysts deposited on carbon paper before electrochemical reaction.** (a-d) Representative images at varying magnifications demonstrate particle sizes ranging from hundreds of nanometers to several micrometers.


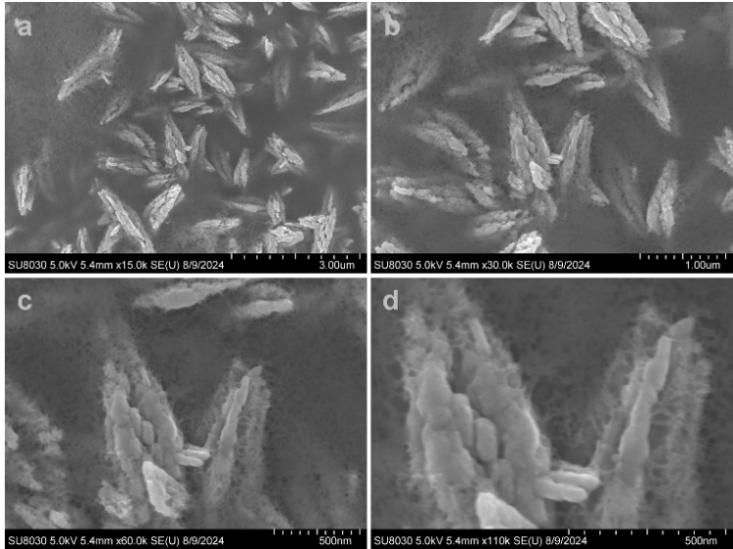


**Supplementary Fig. 25 SEM characterization of CuZn catalysts deposited on carbon paper after electrochemical reaction.** (a-d) Representative images at varying magnifications demonstrating maintained morphological stability after electrochemical testing.


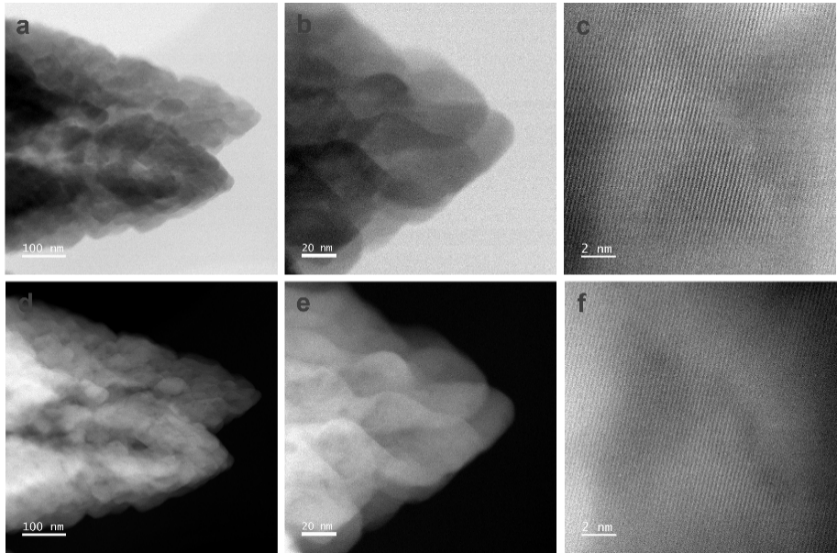


**Supplementary Fig. 26 TEM characterization of CuZn catalysts deposited on carbon paper before electrochemical reaction.** (a-b) Low-magnification TEM images, (c) High-resolution TEM image, (d-f) Corresponding HAADF-STEM images (d→a, e→b, f→c).


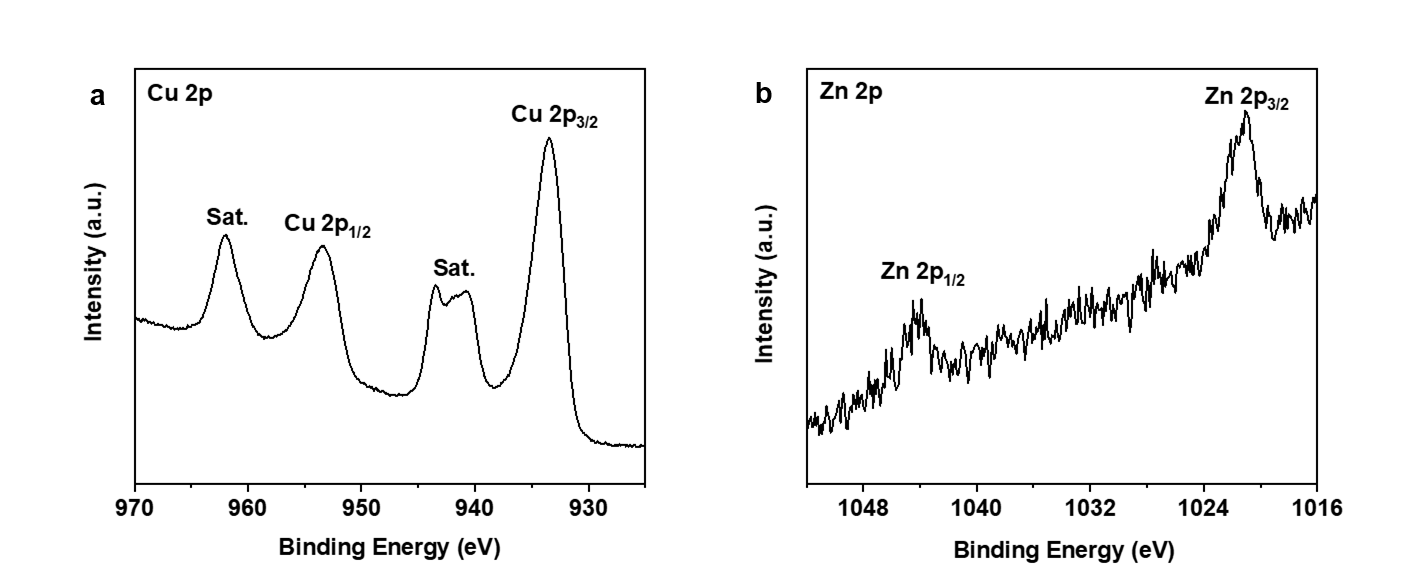


**Supplementary Fig. 27 XPS analysis of CuZn catalyst on carbon paper before electrochemical reaction.** (a) Cu 2p spectrum showing the 2p₃/₂ peak at 933.6 eV with satellite features, (b) Zn 2p spectrum. The observed Cu 2p₃/₂ binding energy (933.6 eV) and characteristic satellite peaks confirm the oxidized state of Cu in CuZn catalyst before reaction. ^[1]^


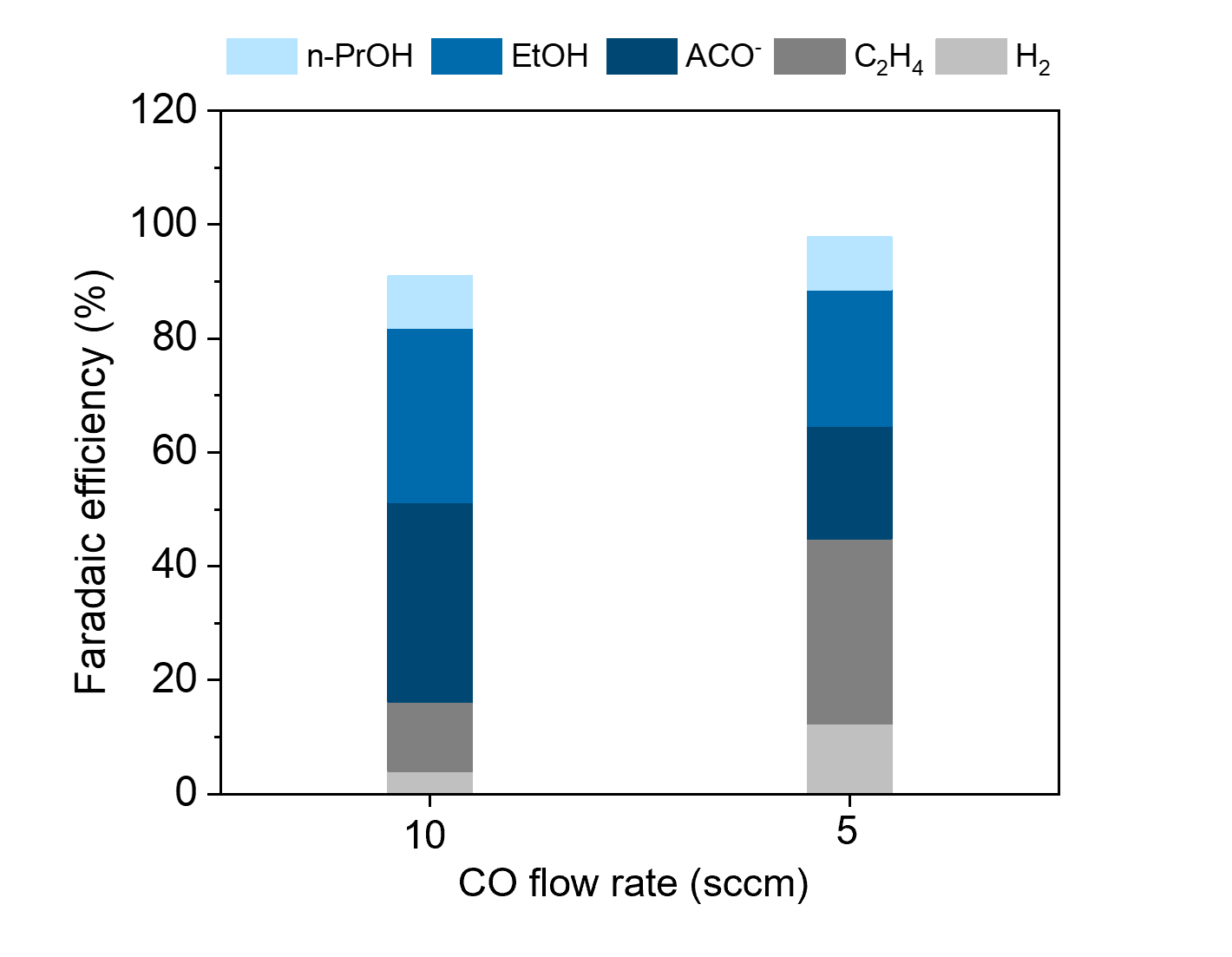


**Supplementary Fig. 28 CO flow rate dependence of COR performance for CuZn catalyst.** When the CO flow rate decreased from 30 sccm (as indicated in the main manuscript), to 10 sccm and 5 sccm, the FE of total gaseous products (H_2_ and C_2_H_4_) increased. This indicates CO mass transport effects on CuZn performance: reducing CO flow rate from 30 → 10 → 5 sccm enhances gaseous product FE by increasing *H coverage (promoting H₂) and lowering *CO coverage (favoring C₂H₄ over C₂₊OH).

**Supplementary Fig. 29 Stability test of CuZn catalyst in K^+^-FB-BPM system.** Test conditions: constant current density of -100 mA/cm^2^ with 1 M KOH anolyte. Over the initial 6 hours, Faradaic efficiency for total gaseous products and total liquid products remained stable, while the cell voltage increased from -2.3 V to -2.5 V after 5 hours. We attribute this voltage shift to K⁺ depletion in the anolyte, as subsequent KOH replenishment restored the cell voltage to -2.3 V.

Comparative analysis: CuSn catalyst showed similar voltage behavior during the stability test at -100 mA/cm^2^ in K⁺-FB-BPM system (Figure 4c)_._ Based on those observations of CuZn and CuSn, we propose a unified ion/molecule transport mechanism for K⁺-FB-BPM system: Continuous K⁺ migration from the anode to the cathode. KOH generation at the AEL/CEL junction, followed by substantial co-transport of KOH and H₂O to the cathode: partial KOH retention on catalyst surface and majority evacuation via cathode gas outlet (with CO). More evidence on this proposal are included in Supplementary Fig. 35.

**Supplementary Fig. 30 COR performance of optimized CuZn catalyst in K^+^-FB-BPM system.** Test conditions: constant current density of -100 mA/cm^2^ with 1 M KOH anolyte. Compared to the CuZn catalyst in manuscript, here the ratio of Zn increased to 2 wt%.

**Supplementary 11. Materials Characterization and COR Performance of CuSn Catalyst in K^+^-FB-BPM System**


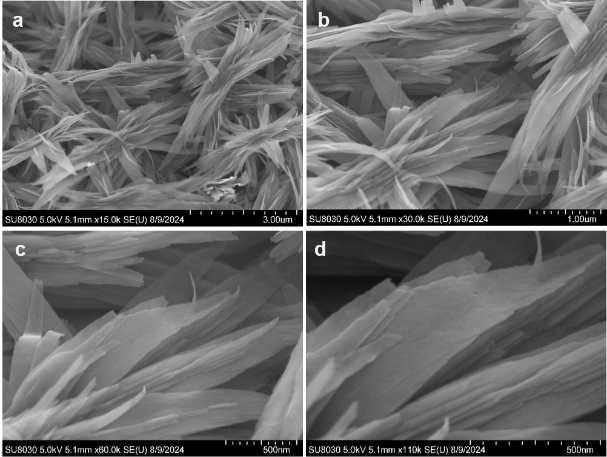


**Supplementary Fig. 31 SEM characterization of CuSn catalysts on carbon paper before electrochemical reaction.** (a-d) SEM images at different magnifications reveal a particle size distribution ranging from hundreds of nanometers to several micrometers.


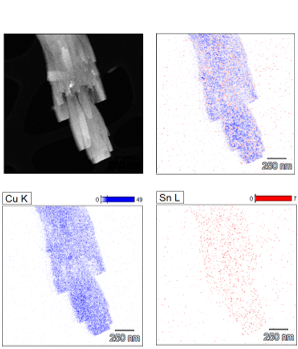


**Supplementary Fig. 32 TEM characterization of CuSn catalysts before reaction.** HAADF-STEM images, corresponding energy-dispersive X-ray spectroscopy (EDS) elemental mapping demonstrating the uniform distribution of Cu (blue) and Sn (red).


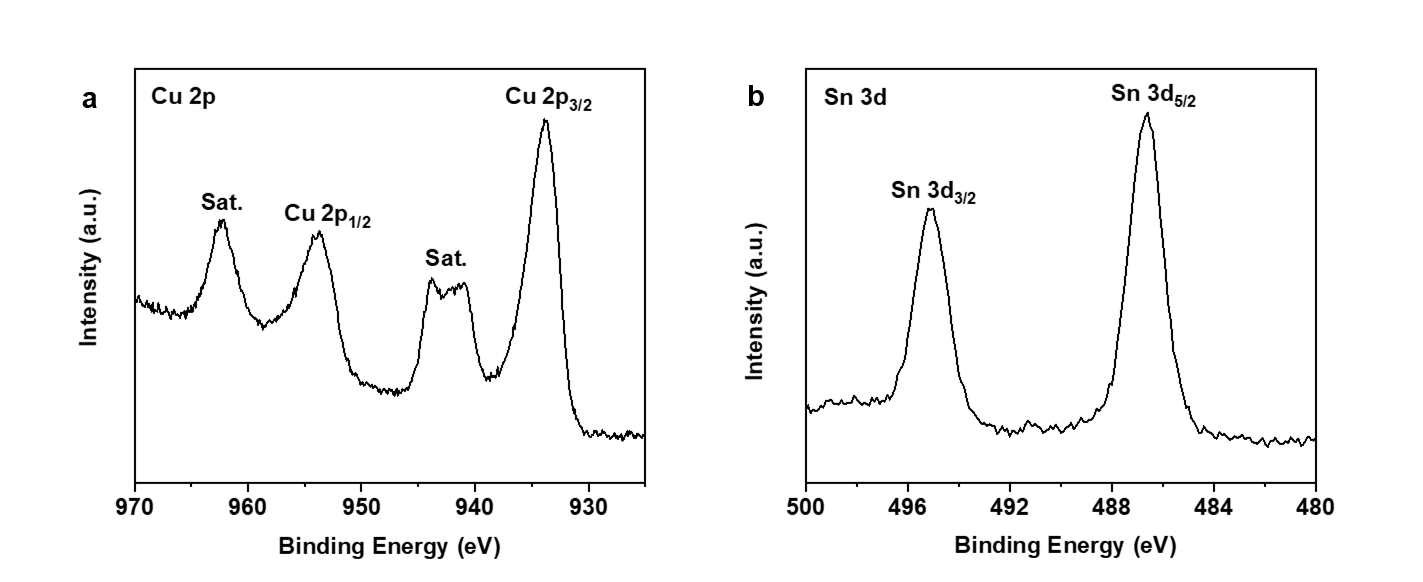


**Supplementary Fig. 33 XPS analysis of CuSn catalyst on carbon paper**. (a) Cu 2p spectrum exhibiting 2p₃/₂ peak at 933.6 eV and distinct satellite features, (b) Sn 3d. The observed Cu 2p₃/₂ binding energy (933.6 eV) with characteristic satellites confirms the presence of Cu²⁺ species.


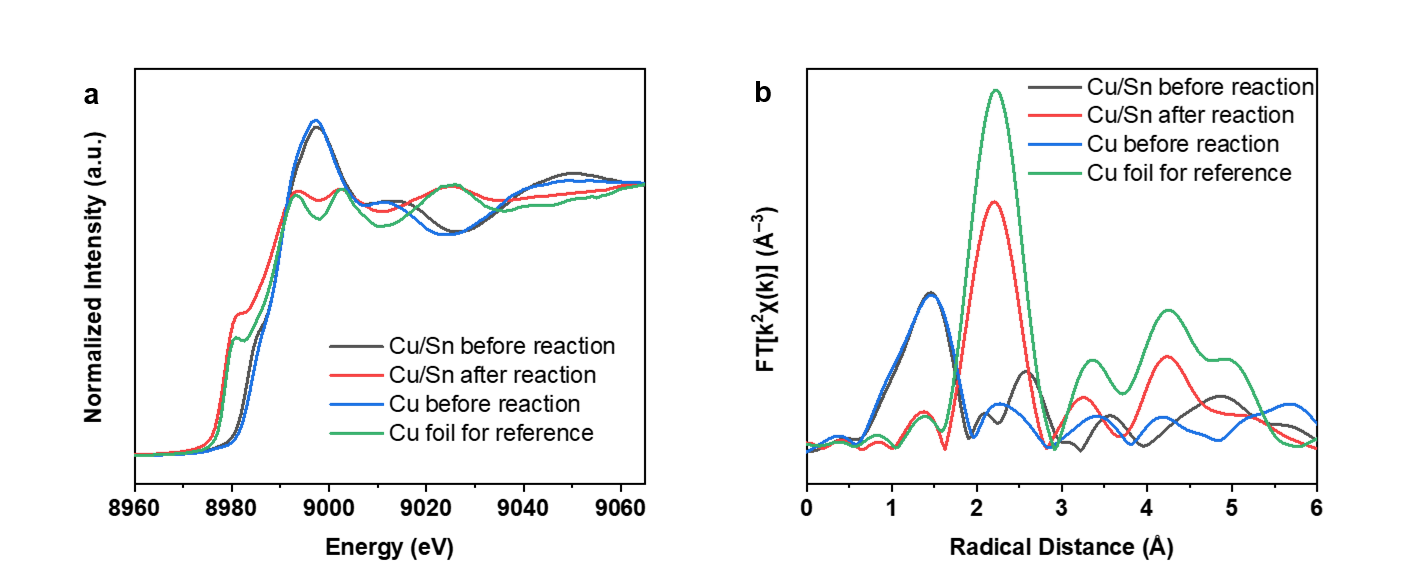


**Supplementary Fig. 34 Ex-situ X-ray absorption spectroscopy (XAS) measurement at the Cu K-edge for CuSn catalyst, Cu catalyst and referenced Cu foil.** (a) X-ray absorption near-edge structure (XANES) spectra, highlighting changes in the oxidation state of Cu species of the Cu K-edge of catalysts. (b) Extended X-ray absorption fine structure (EXAFS) spectra, illustrating the local coordination environment of Cu atoms of Cu K-edge in catalysts. Ex-situ XAS reveals that the Cu species in CuSn catalysts are initially oxidized prior to the CO reduction reaction (CORR), but are reduced to a nearly metallic Cu state after CORR, as evidenced by the XANES, which closely resemble those of the reference Cu foil. From the EXAFS data, both pristine CuSn and pristine Cu exhibit a prominent peak at approximately 1.5 Å, corresponding to Cu-O coordination. Notably, only the CuSn sample shows a relatively strong peak at around 2.6 Å, attributable to Cu-Cu/Sn coordination. This observation suggests that the pristine CuSn catalyst may possess a more crystalline oxide phase with a larger particle size compared to pristine Cu. ^[1, 9]^

**Supplementary 12. Investigations on the Concentration of K^+^ and OH^-^ for CuSn Catalyst in K^+^-FB-BPM System**


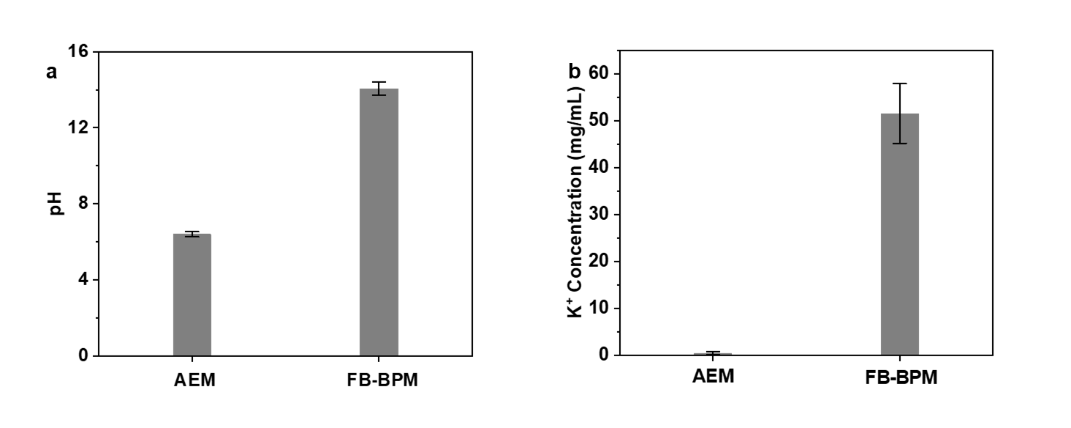


**Supplementary Fig. 35 (a) pH and (b) K^+^ concentration profiles for CuSn catalyst in** **K^+^-AEM system and K^+^-FB-BPM system.** Test conditions: Constant current densities of -100 mA/cm^2^ with 1 M KOH anolyte. The samples collection points are as specified in main text (Figure 3f). Cathodic liquid products were dispersed in 2 mL deionized water for pH measurement, yielding: the pH of 14.1 for K^+^-FB-BPM system and the pH of 6.3 for K^+^-AEM system. The detected K^+^ concentration at the cathodic side in K^+^-FB-BPM system was around 100 times higher than that of K^+^-AEM system (The inaccuracy in measuring extremely low K^+^ concentration may affect the absolute difference of K^+^ concentration between K^+^-FB-BPM system and K^+^-AEM system). Although it’s previously reported that the increased OH^-^ promotes H_2_ suppression and enhanced C_2_H_4_ generation^[10]^, our experimental observations show the existence of K^+^ and extremely high OH^-^ concentration are generally favorable for H_2_ suppression and enhanced C2+ liquid products generation. The possible speculation is that when OH^-^ concentration at cathode is moderately high, it’s favorable for H_2_ suppression and for enhanced C_2_H_4_ generation, however, when OH^-^ concentration at cathode is extremely high, it would be favorable for both H_2_ suppression and C_2_H_4_ suppression while C2+ liquid products become the main product.

**References**

[1] X. Wang et al. Site-selective protonation enables efficient carbon monoxide electroreduction to acetate. *Nature Communications* **2024**, *15*, 616.

[2] S. Overa et al. Enhancing acetate selectivity by coupling anodic oxidation to carbon monoxide electroreduction. *Nature Catalysis* **2022**, *5*, 738-745.

[3] W. Kohn and L. J. Sham. Self-Consistent Equations Including Exchange and Correlation Effects. *Physical Review* **1965**, *140*, A1133-A1138.

[4] a) G. Kresse and D. Joubert. From ultrasoft pseudopotentials to the projector augmented-wave method. *Physical Review B* **1999**, *59*, 1758-1775; b) G. Kresse and J. Furthmüller, Efficient iterative schemes for ab initio total-energy calculations using a plane-wave basis set. *Physical Review B* **1996**, *54*, 11169-11186.

[5] P. E. Blöchl, Projector augmented-wave method. *Physical Review B* **1994**, *50*, 17953-17979.

[6] a) J. P. Perdew, K. Burke and M. Ernzerhof, Generalized Gradient Approximation Made Simple. *Physical Review Letters* **1996**, *77*, 3865-3868; b) J. P. Perdew et al. Atoms, molecules, solids, and surfaces: Applications of the generalized gradient approximation for exchange and correlation. *Physical Review B* **1992**, *46*, 6671-6687.

[7] a) S. Grimme, J. Antony, S. Ehrlich, H. Krieg. A consistent and accurate ab initio parametrization of density functional dispersion correction (DFT-D) for the 94 elements H-Pu. *The Journal of Chemical Physics* **2010**, *132*, 154104; b) S. Grimme, S. Ehrlich and L. Goerigk, Effect of the damping function in dispersion corrected density functional theory. *Journal of Computational Chemistry* **2011**, *32*, 1456-1465.

[8] a) K. Mathew, V. S. C. Kolluru, S. Mula, S. N. Steinmann, R. G. Hennig, Implicit self-consistent electrolyte model in plane-wave density-functional theory. *The Journal of Chemical Physics* **2019**, *151,* 234101; b) K. Mathew, R. Sundararaman, K. Letchworth-Weaver, T. A. Arias and R. G. Hennig, Implicit solvation model for density-functional study of nanocrystal surfaces and reaction pathways. *The Journal of Chemical Physics* **2014**, *140,* 084106.

[9] a) J. Jin et al. Constrained C2 adsorbate orientation enables CO-to-acetate electroreduction. *Nature* **2023**, *617*, 724–729; b) J. E. Huang et al. CO_2_ electrolysis to multicarbon products in strong acid. *Science* **2021**, *372*, 1074–1078.

[10] a) C. Kim et al. Tailored catalyst microenvironments for CO_2_ electroreduction to multicarbon products on copper using bilayer ionomer coatings. *Nature Energy* **2021**, *6*, 1026–1034; b) D. Cheng et al. Guiding catalytic CO_2_ reduction to ethanol with copper grain boundaries. *Chemical Science* **2023**, *14*, 7966.
